# Analysis of the hybrid proline-rich protein families from seven plant species suggests rapid diversification of their sequences and expression patterns

**DOI:** 10.1186/1471-2164-8-412

**Published:** 2007-11-12

**Authors:** Lenka Dvořáková, Fatima Cvrčková, Lukáš Fischer

**Affiliations:** 1 Charles University in Prague, Faculty of Science, Department of Plant Physiology, Viničná 5, 128 44 Prague 2, Czech Republic

## Abstract

**Background:**

Plant hybrid proline-rich proteins (HyPRPs) are putative cell wall proteins consisting, usually, of a repetitive proline-rich (PR) N-terminal domain and a conserved eight-cysteine motif (8 CM) C-terminal domain. Understanding the evolutionary dynamics of HyPRPs might provide not only insight into their so far elusive function, but also a model for other large protein families in plants.

**Results:**

We have performed a phylogenetic analysis of HyPRPs from seven plant species, including representatives of gymnosperms and both monocot and dicot angiosperms. Every species studied possesses a large family of 14–52 HyPRPs. Angiosperm HyPRPs exhibit signs of recent major diversification involving, at least in *Arabidopsis *and rice, several independent tandem gene multiplications. A distinct subfamily of relatively well-conserved C-type HyPRPs, often with long hydrophobic PR domains, has been identified. In most of gymnosperm (pine) HyPRPs, diversity appears within the C-type group while angiosperms have only a few of well-conserved C-type representatives. Atypical (glycine-rich or extremely short) N-terminal domains apparently evolved independently in multiple lineages of the HyPRP family, possibly via inversion or loss of sequences encoding proline-rich domains. Expression profiles of potato and *Arabidopsis HyPRP *genes exhibit instances of both overlapping and complementary organ distribution. The diversified non-C-type *HyPRP *genes from recently amplified chromosomal clusters in *Arabidopsis *often share their specialized expression profiles. C-type genes have broader expression patterns in both species (potato and Arabidopsis), although orthologous genes exhibit some differences.

**Conclusion:**

HyPRPs represent a dynamically evolving protein family apparently unique to seed plants. We suggest that ancestral HyPRPs with long proline-rich domains produced the current diversity through ongoing gene duplications accompanied by shortening, modification or loss of the proline-rich domains. Most of the diversity in gymnosperms and angiosperms originates from different branches of the HyPRP family. Rapid sequence diversification is consistent with only limited requirements for structure conservation and, together with high variability of gene expression patterns, limits the interpretation of any functional study focused on a single *HyPRP *gene or a couple of *HYPRP *genes in single plant species.

## Background

Hybrid proline-rich proteins (HyPRPs) represent a typical example of a protein family characterized by well-defined sequence features but little functional knowledge besides a loosely defined role in the development or function of the plant cell wall. Since a large part of the cellular molecular machinery is conserved across multiple kingdoms, characterization of truly lineage-specific gene families involved in lineage-specific biological processes or structures may provide clues for grasping the fundamentals of the current diversity of organisms. Moreover, understanding molecular mechanisms of plant cell wall evolution, ontogeny, and function, is of more than purely theoretical interest. This is because cell wall properties substantially contribute to the physical properties of plant tissues, which are of central importance in almost all areas of human activity concerned with plant materials (such as food or fiber processing).

HyPRPs are characterized by the presence of two different domains: a proline-rich N-terminal repetitive domain and a hydrophobic C-terminal domain. Based on the proline-rich domain and a secretory signal, HyPRPs belong to the group of secreted structural cell wall proline-rich proteins [1]. The repetitive character and high proline content of N-terminal domains resemble other proline-rich proteins, though the repeated amino acid motifs vary [2]. The hydrophobic C-terminal domain classifies HyPRPs into the group of proteins containing the 8 CM domain [3], together with lipid transfer proteins (LTPs), amylase inhibitors, 2S albumins and some other subgroups. The characteristic 8 CM domain usually consists of less than 100 amino acid residues and contains eight cysteine residues in a specific order [3]. Tertiary structure formed by four hydrophobic helices stabilized by the conserved cysteines has been determined for several proteins of the family since the first reports on crystallographic analysis of the soybean (*Glycine max*) hydrophobic seed protein [4]) and NMR analysis of wheat LTP in solution [5]. Although no structural data are available for proteins of the HyPRP subgroup, which has been, so far, analyzed only on the sequence level, we can assume that the tertiary structure of their C-terminal domains resembles other 8 CM proteins. However, the conserved structure does not allow any functional predictions. It apparently serves only as a scaffold carrying specific functional elements in various subgroups of the 8 CM family [2].

Multiple reports indicated variable patterns of *HyPRP *gene expression, but the studies mostly focused on a single or a few genes. In alfalfa (*Medicago sativa*), expression of *MsPRP2 *was induced by water deficit in salt-tolerant plants [6], while *MsACIC *was transcribed in cold-tolerant plants [7]. *BNPRP *from *Brassica napus *was also highly expressed at low temperature. However, low levels of the *BNPRP *transcript were also detected at standard growth conditions [8]. Expression of *SbPRP *from soybean was modulated by ABA, internal circadian rhythm and some stress factors. The expression was induced in response to viral infection or by salicylic acid treatment [9]. The transcript of *CrHyPRP *was detected in subapical stem segments of *Cuscuta reflexa *that were sensitive to the induction of haustoria formation by cytokinins [10]. Gene *DC 2.15 *from carrot (*Daucus carota*) was down regulated by auxin and its expression was detected in vascular bundles, leaves and flower discs [11]. The *FaHyPRP *gene from strawberry (*Fragaria ananassa*) was specifically induced in mature fruits [12]. *MtPPRD1 *from barrel medic (*Medicago truncatula*) was specifically expressed in the axial part of the embryo during germination and constitutively expressed in roots [13]. Maize (*Zea mays*) *ZmHYPRP1 *transcript accumulated in parenchyma cells of the embryo, but was undetectable in other adult plant organs except the ovary prior to pollination [14]. The transcript of the *CELP *gene family of tobacco (*Nicotiana tabacum*) was specifically accumulated in flower organs [15].

The expression of *HyPRP *genes can vary even between highly similar proteins in closely related species. The gene encoding tomato (*Lycopersicum esculentum*) TPRP-F1 was expressed almost exclusively in immature fruits [16]. However, expression of the closely related *StPRP *from potato (*Solanum tuberosum*) was not detected in the potato berry, whereas a high transcript level was detected in roots [17].

Together, these data show that expression of *HyPRP *genes is highly variable. Transcripts of different genes were detected in various stages of plant development and under diverse conditions. Although the *HyPRP *genes have never been proven to play a specific role in any biological process, the genes were found in numerous screens, probably due to high absolute transcript levels under certain inductive conditions or at specific developmental stages.

In this paper, we present a comparative study of the HyPRP families of potato and *Arabidopsis thaliana*. We have compared experimentally determined expression patterns of 14 potato *HyPRP *genes with publicly available expression data for their orthologs and paralogs from the *Arabidopsis *Genevestigator database [18]. In order to gain a better understanding of the evolutionary dynamics of the plant *HyPRP *gene family, we have performed a detailed phylogenetic analysis of amino acid sequences of all available *HyPRP *genes sequences from additional five seed plant species. This analysis included representatives of both closely related (dicot) and less closely related (monocot and gymnosperm) groups.

## Results

### Inventory and expression patterns of potato *HyPRP *genes

We initially found sequences of fourteen potato cDNAs encoding HyPRPs (further referred to as St1 to St14) in the public SOL Genomics Network (SGN) database (see Methods). These were used to design specific primers for semiquantitative RT-PCR estimation of transcript levels in plant tissues. Subsequently, we identified two additional genes (a partial sequence denoted St15, and St16) in the database and included them in the bioinformatic and phylogenetic study, although they have not been followed experimentally (see Table 1 for a list of genes and primer sequences).

**Table 1:** Genes encoding HyPRPs in potato

**Gene**	**Accession number**	**Primers**	**Product (bp)**
**St1**	SGN-U268850	GGT GGA AGT GCT AAG CAA ACA	189
		GGT TGA AGG ACA CTT GAA GTC	
**St2**	SGN-U268572	TTG GGC TTG GTG ACC CAG C	192
		ATG GAG CAA GTG TAG CCA GG	
**St3**	SGN-U269819	TGT TAT TGG AAG TAG CCC AGC	225
		GTG GAG AAA TTT GGC TAT AGC A	
**St4**	SGN-U271014	TTG GAG TTG TAC TTG GAA ATC C	269
		CGA AGA TTC ATT ATA GCT GAC C	
**St5**	SGN-U269818	TTG TTA TAG GAA GTA GCC CAA C	216
		GAA AGA AAC TAA ATT TAT CTT AAG C	
**St6**	SGN-U278851	TGC ATG TTG TCA TTG GAA GCC	237
		AGA AGA AAC ACA GAA ATG GTT TG	
**St7**	SGN-U272247	TAA ATG TAA CAC TTG GCA CTC C	234
		ATG AAT ATC AAA AAC ACA AAA GGC	
**St8**	SGN-U288400	TAA AAT GTA ACA CTT GGA ACT CC	237
		TAA ATG TAG AAG CAA ACT CAA CTA	
**St9**	SGN-U269258	AGT TAA TGT TGT TGT TGG TTC AC	244
		AAA AGT CTA CAC AGA AAG ATC GA	
**St10**	SGN-U272649	TCG GAG CGG TCA TTG GGA C	189
		AAA TCA GAT GGG AGT GTT TTG C	
**St11**	SGN-U269259	AGT AAA TGT TAT TGT TGG CTC AC	253
		ACC CCT CAA CCT CAA AGG AC	
**St12**	SGN-U274282	ATT GGG GCA AAA CCA AGT AGC	215
		GCG TAA AAT CCT GTA TAC GCC	
**St13**	SGN-U276378	GTT GCA ATT GGT AGC CAA GTG	187
		AAA CCT GTG GGA ACT TTC TTA G	
**St14**	SGN-U276758	CTT AAC GTG GTG AAT GTA ACA G	224
		ATT AGG AGG GTA GTT TAA CAA GT	
**St15**	SGN-U279209	-	-
**St16**	SGN-U272246	-	-

Accession numbers, primer sequences (both from 5' to 3', forward primer first) and predicted length of PCR products.

**Figure 1:**
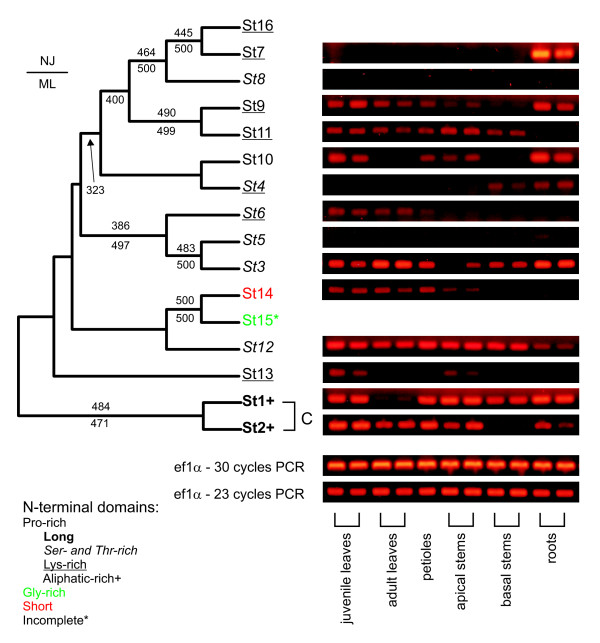
**Evolutionary relationships and expression patterns of potato *HyPRPs***. Left: an unrooted consensus phylogenetic tree (cladogram) of nucleotide sequences encoding the conserved C-terminal domains of HyPRPs from potato (*Solanum tuberosum*), constructed by the NJ method using programs from the PHYLIP package (see Materials and Methods). Bootstrap values above 50 % (from 500 replicates) are shown above the individual branches, numbers below branches denote bootstrap values of a ML tree from the same input data. Gene names are color-coded according to the composition of the N-terminal domains of the encoded proteins (see Table 2). "Standard" proline-rich N-terminal domains (more than 20 % of Pro, Pro to Gly ratio larger than 2) are shown in black, glycine-rich N-terminal domains (more than 20 % of Gly, Gly to Pro ratio larger than 2) in green, N-terminal domains shorter than 10 amino acid residues in red. Among proteins with "standard" (Pro-rich) N-terminal domains, those with extremely long N-termini (over 80 residues) are shown in **bold**, those with increased contents of serine and threonine in *italics*, lysine-rich ones are underlined, hydrophobic (A, V, L, I-rich) are marked by a plus (+) sign. An asterisk denotes a truncated N-terminus. Right: expression profiles of fourteen potato *HyPRP *genes (see Table 1) in vegetative organs of *in vitro *cultured potato plants determined by semiquantitative PCR. Expression of the ef1a gene was used as the internal standard (products of PCR with 23 and 30 cycles are shown).

All identified potato HyPRPs, except St15 (which is apparently N-terminally truncated), possess an unambiguous N-terminal secretion signal. The following N-terminal domains are predominantly repetitive, with alkaline character (isoelectric point around 8.5–10) resulting mainly from higher content of lysine and histidine. Otherwise, the N-terminal domains are highly variable with respect to size, amino acid composition and repetitive sequence motifs (Table 2). However, the 8 CM C-terminal domains are conserved enough to allow multiple alignment construction and phylogenetic analyses. Due to small size of these domains, only a few branches with reasonably high bootstrap values have been identified in trees constructed on the basis of protein sequences. The tree topology varied depending upon the method used (see Additional file 1). However, support for several clades improved dramatically when corresponding nucleotide (cDNA) sequences were taken into account and all the stable clades from the protein sequence-based tree were recovered (Figure 1). Remarkably, the sequences St1 and St2 shared not only a significant degree of similarity, but also some features of their N-terminal domains, which are very long (over 125 residues), with a complex repetitive structure and a high content of aliphatic amino acids (alanine, valine, leucine, and isoleucine). We shall further refer to St1 and St2 (and their relatives from other species – see below) as "C-type" (conserved) HyPRPs. The majority of remaining HyPRPs have N-terminal domains with a very low content of aliphatic residues, usually in the context of short (0–39 residues) proline-rich domains containing di- or tripeptide repeats (consensus pattern P-X or P-S-X). Some similarities could also be identified for several other subgroups of HyPRPs (see Table 2 and Figure 1). In particular, the N-terminal domains of St4, St7, St9, St11 and St16, which cluster together in the C-terminal domain-based tree, have a high content of lysine, usually present in the repetitive motif P-K. The sequences of St3, St5 and St6 also form a stable branch in the tree and all of them have N-terminal domains with a relatively high content of serine and threonine.

**Table 2:** Properties of the N-terminal domains of potato HyPRPs

**Gene**	**N (aa)**	**% AVLI**	**% K**	**% S**	**% T**	**% P**	**Repeats**	**n**
**St16**	26	0	**23,1**	3,8	7,7	42,3	P-[KPTC]	9
**St7**	24	0	**20,8**	4,2	8,3	41,7	P-[KTPYC]	8
**St8**	23	0	8,7	8,7	**13**	39,1	P-[STIKND]	9
**St9**	24	4,2	**20,7**	4,2	4,2	45,8	P-[KPS]	9
**St11**	21	0	**19**	4,8	4,8	42,9	P-[KPS]	8
**St10**	27	3,7	3,7	3,7	3,7	44,4	P-[NSPYK]	11
**St4**	22	0	**22,7**	**13,6**	9,1	40,9	P-[KST]	9
**St6**	13	0	**15,4**	**15,4**	7,7	30,8	P-P-[KS]-T	2
**St5**	28	3,6	3,6	7,1	**21,4**	46,4	P-[THKPC]	9 2
							P-S-[TS]	
**St3**	39	2,6	5,1	**15,4**	**20,5**	41	P-[THPYKC]	8 6
							P-S-[TS]	
**St14**	3	very short N-terminal domain					no repeats	
**St15**	18	incomplete – polyG fragment					no repeats	
**St12**	11	9,1	9,1	0	**18,2**	36,4	P-[STPC]	4
**St13**	19	10,6	**15,8**	0	5,3	42,1	P-[KTPIYHC]	8
							P-K-H-P-K-[LY]-P	2
**St1**	246	**19,1**	6,9	6,1	9,3	46,7	P-[ISVYPKH]-[VPYK]-[SKHTYVIQ]-P	25
							P-[IVFH]-[TVI]-P-[TKRN]-P	11
**St2**	128	**32**	10,9	2,3	3,1	37,5	P-[IV]-[GVDTHI]-[KVLI]-P	19

Proteins are ordered according to their position in the phylogenetic tree of C-terminal domains (see Figure 1). Domain length N (from the predicted end of the signal peptide to the first cysteine of the 8 CM domain), content of selected amino acids (%), PROSITE patterns of the repetitive units (with amino acids at variable positions ordered according to frequency), and numbers of repeats (n). Values typical for proteins from individual branches shown in bold. The sequence of St15 is truncated.

Fourteen originally identified potato *HyPRP *genes were analyzed with respect to their expression in vegetative organs of *in vitro *grown potato plants – young apical leaves, mature leaves, petioles, growing apical stems, basal stems and roots (Figure 1). In every organ studied, each major subgroup of HyPRPs was represented by at least one expressed gene. While some genes were expressed more or less exclusively in certain organs (e.g. St7 in roots), others exhibited high expression levels in many (or all) organs tested (e.g. St3 or St12). Divergent and, sometimes, complementary patterns of expression of closely related genes have been observed (in particular for the St3/St5, St1/St2, and St11/St9 pairs). For instance, St1 expression is absent in mature leaves, where St2 is expressed, while the opposite holds for basal stems.

### Comparison of the potato and *Arabidopsis HyPRP *gene families

Since the potato genome is only partially sequenced, our inventory of *HyPRP *genes is probably incomplete, although highly expressed genes are likely to be included. However, the *Arabidopsis *genome has been completely sequenced and extensively annotated. A large set of transcriptome data is available for this model organism, providing information about at least one full set of dicotyledonous plant HyPRPs.

**Figure 2:**
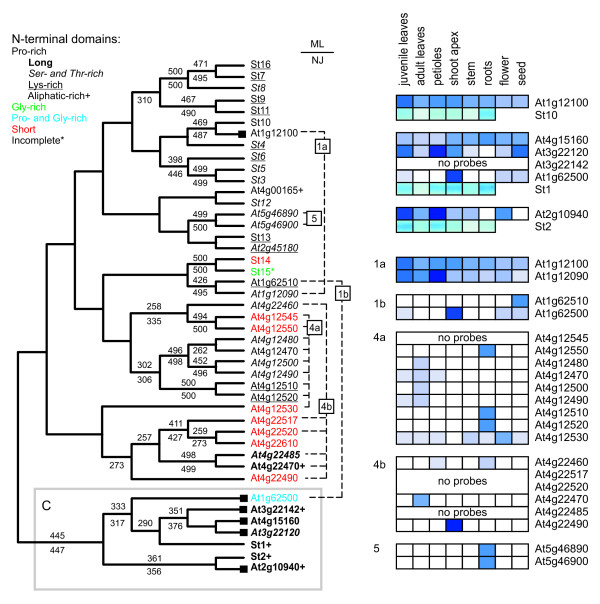
**The HyPRP families of potato and *Arabidopsis*. **Left: an unrooted consensus phylogenetic tree (cladogram) of nucleotide sequences encoding the conserved C-terminal domains of HyPRPs from potato and *Arabidopsis thaliana*, constructed by the ML method (see Materials and Methods). Bootstrap values above 50 % (from 500 replicates) are shown above the individual branches, numbers below branches denote bootstrap values of a NJ tree from the same input data. The ML and NJ trees agreed in all clades with bootstrap support over 50 % with exception of (i) swapping St1 and At1g62500 and (ii) swapping At4g15160 and At4g15160, in both cases with NJ bootstrap values below 76 %. *Arabidopsis *loci are denoted by standard AGI locus identifiers. Gene names are color-coded and typographically marked according to the composition of the N-terminal domains of the encoded proteins as in Figure 1; in addition, proline- and glycine-rich N-terminal domains (not fitting into the categories of proline- or glycine-rich, but containing more than 10 % of each of these amino acids) are shown in blue. *Arabidopsis *genes with reliable potato orthologues are denoted by black filled squares, chromosomal clusters and tandem duplications are marked by chromosome cluster numbers to the right of the tree (see Additional file 2). Right: expression profiles of selected *Arabidopsis HyPRP *genes according to the publicly available expression data: top – orthologues of potato genes (with potato expression patterns from Figure 1 for comparison; apical stems shown in the "shoot apex" position), bottom – genes from the four *Arabidopsis *chromosomal clusters. Order of genes within each cluster corresponds to that in the tree.

Our BLAST searches of *Arabidopsis *genome sequences revealed the presence of 28 HyPRP-encoding loci (Figure 2). This included all 23 genes reported previously by José-Estanyol and colleagues [2], who also already noticed that the majority of *Arabidopsis *HyPRPs are encoded by genes residing in clusters of tandem duplications. We could identify five such sets of immediately or nearly immediately adjacent *HyPRP *loci: two pairs on chromosome 1 (referred to as clusters 1a and 1b), two clusters on chromosome 4 (cluster 4a, consisting of 9 genes and cluster 4b with 6 genes) and a pair on chromosome 5 (cluster 5b). Remaining genes are located on chromosome 2 (2 loci), 3 (2 loci relatively close to each other) and 4 (2 isolated loci and one gene close to cluster 4b). A common phylogenetic analysis of *Arabidopsis *and potato sequences encoding the conserved C-terminal domain grouped together a majority of *Arabidopsis *sequences from chromosomal cluster 4a with each other and cluster 4b with each other, as well as the pair from chromosome 5 (cluster 5). This suggests relatively recent gene duplication events. However, the immediately neighboring sequences from pairs 1a and 1b appear to be substantially divergent, indicating either ancient gene duplication or gene conversion, i.e. exchange of a closely related gene for its distant relative. As in potato, we found several groups of genes with high bootstrap values. Most notably, this was a branch of HyPRPs sharing not only mutually related C-terminal domains, but also long and complex N-terminal domains rich in hydrophobic amino acid sequences. This branch also contains both potato C-type HyPRPs (St1 and St2), indicating that these proteins indeed deserve to be considered a specific HyPRP type. As in the case of potato, we could recognize similarities in amino acid composition or common sequence motifs in the N-terminal domains of proteins located in several branches of the tree, although the N-termini are apparently less conserved than the corresponding C-terminal domains.

To our surprise, we were barely able to find clearly defined *Arabidopsis *orthologues of most of the potato genes. Besides the two C-type genes (St1 and St2), only St10 had a close relative with a significant bootstrap support. Moreover, 8 of the 16 potato *HyPRP *genes clustered into three independent branches devoid of *Arabidopsis *sequences (a branch containing St7, St8, and St16; a branch containing St9 and St11; and a branch containing St3, St5 and St6). This indicates that major diversification of the *HyPRP *gene family took place after separation of the lineages leading towards Solanaceae and Brassicaceae.

This hypothesis is also supported by analyses of the available expression data, which revealed no clear relationship between expression patterns of orthologous *Arabidopsis *and potato genes. In contrast, paralogous genes from the *Arabidopsis *chromosomal clusters (especially 4a and 4b) shared specialized expression patterns (Figure 2), which is also consistent with their recent origin. Apparently, C-type genes are predominantly transcribed in almost all analyzed organs in both *Arabidopsis *and potato, while expression of most of the remaining genes is limited to one or a few organs.

### Phylogenetic analysis of aminoacid sequences of *HyPRP *genes from seven plant species

**Figure 3:**
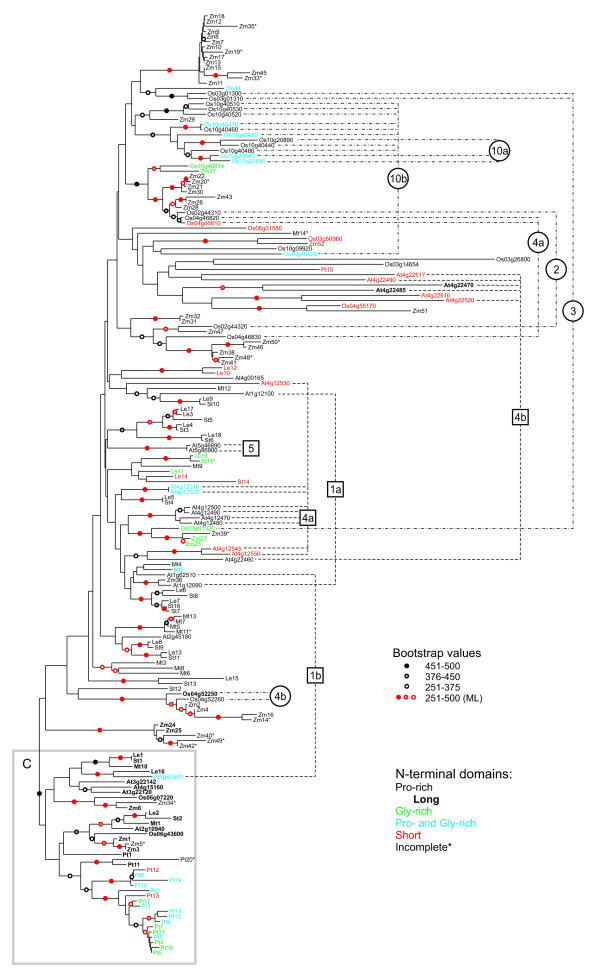
**Phylogeny of plant HyPRPs**. An unrooted phylogenetic tree, constructed by the NJ method using Treecon (see Materials and Methods) from amino acid sequences of C-terminal domains of HyPRPs from seven plant species (At – *Arabidopsis thaliana*, Le – *Lycopersicum esculentum*, Mt – *Medicago truncatula*, Os – *Oryza sativa*, Pt – *Pinus taeda*, St – *Solanum tuberosum*, Zm – *Zea mays*). *Arabidopsis *loci are denoted by standard AGI locus identifiers, rice loci by a shortened version of the TIGR Rice genome annotation identifiers (i.e. Os02g44310 stands for LOC_Os02g44310 etc.). Symbols at branches denote bootstrap values (out of 500 replicates), red symbols denote branches which had bootstrap support over 50 % also in a ML tree from the same input data. The NJ and ML trees agreed in all clades with bootstrap support over 50 % with exception of the ML tree (i) swapping Pt2 and Pt13, (ii) clustering together Zm25 and Zm40, (iii) clustering together Mt11 an Mt5, (iv) clustering Zm 19 with the Zm33/Zm45 pair, and (v) locating Os04g46830 into the cluster containing Zm47. Sequences are color-coded as in Figures 1 and 2: "Standard" proline-rich N-terminal domains are shown in black (long ones in **bold**), glycine-rich N-terminal domains in green, proline- and glycine-rich N-terminal domains in blue, N-terminal domains shorter than 10 amino acid residues are in red, truncated N-termini are denoted by an asterisk; chromosomal clusters and tandem duplications in *Arabidopsis *(squares) and rice (circles) are marked to the right of the tree. Chromosomal clusters are numbered according to their chromosomal positions (i.e. 10a and 10b means the two clusters on rice chromosome 10).

The above-described analysis of the 16 potato and 28 *Arabidopsis HyPRP *genes revealed unexpected differences between the two species. We therefore decided to compare these results with data from two additional dicotyledonous species, barrel medic (*Medicago truncatula*) and tomato (*Lycopersicon esculentum*), another member of Solanaceae family. We also carried out a comparison with representatives of monocotyledonous plants, rice (*Oryza sativa*), and maize (*Zea mays*) and a gymnosperm, loblolly pine (*Pinus taeda*). We could not find any *bona fide *HyPRP sequences in non-seed plants (algae, mosses and ferns; for criteria see Materials and Methods). However, in all angiospermous and gymnospermous species studied, HyPRPs form large families. Our database searches revealed 19 complete or nearly complete *HyPRP *cDNA sequences from tomato, 14 from medic, 52 from maize and 21 from pine. Thirty-one well-defined HyPRP-encoding loci (containing a putative secretory signal and a C-terminally located 8 CM domain that could be well aligned to the potato and *Arabidopsis *sequences) were found in the fully sequenced rice genome. Several other genes that lacked parts of the conserved 8 CM domain were excluded from further analyses (the total number of rice genes carrying some variant of the 8 CM domain exceeds sixty). Most notably, some of the rice *HyPRP *genes occurred in pairs or clusters of closely adjacent loci (pairs on chromosomes 2 and 4, triplets on chromosomes 3, 4 and 10 and a cluster of 9 genes on chromosome 10). This shows that rapid and recent amplification of *HyPRP *genes is obviously not restricted to *Arabidopsis *(see Figure 3).

**Figure 4:**
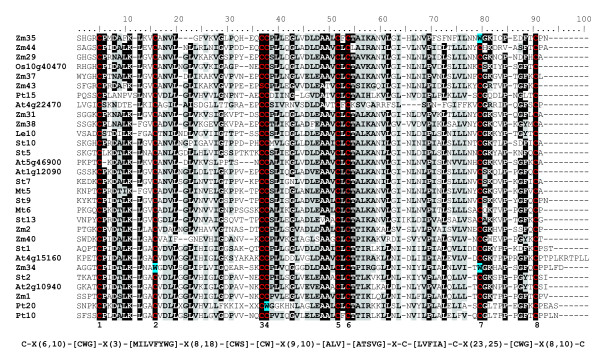
**The conserved C-terminal 8 CM domains of selected plant HyPRPs. **Top: multiple alignment of the conserved C-terminal domain sequences from representative HyPRPs of all major phylogenetic branches (see Figure 3). At – *Arabidopsis thaliana*, Le – *Lycopersicon esculentum*, Mt – *Medicago truncatula*, Os – *Oryza sativa*, Pt – *Pinus taeda*, St – *Solanum tuberosum*, Zm – *Zea mays*. Residues conserved between at least 75 % of the depicted sequences are shown on black background, positions with conserved amino acid properties on gray background. Conserved cysteines are shown in red and denoted by numbers, selected substitutions at the conserved cysteine positions are highlighted in blue. Bottom: consensus sequence expressed as a PROSITE – style pattern that detects most *Arabidopsis *HyPRPs and no false positives.

Our subsequent phylogenetic study was based on a collection of 181 sequences (see Additional file 2). As in the cases of potato and *Arabidopsis*, N-terminal domains of HyPRPs from other species are also highly variable. A multiple alignment of conserved C-terminal domains (Figure 4, Additional file 3), which was used to calculate a phylogenetic tree (Figure 3), revealed that only relatively few amino acid residues are absolutely conserved (i.e., invariant among all sequences sampled). Surprisingly, even one or two of the eight cysteines characteristic for the 8 CM domain have been replaced by other residues (usually tryptophan) in 16 HyPRPs; 10 of these were from maize. These substitutions most frequently affected cysteines 2 and 5 of the 8 CM motif (see Figure 4); however, nearly every one of the conserved cysteines was replaced by another amino acid at least in one of the sequences. Chemical properties of multiple amino acids were conserved, although we could not identify any specific positions or regions within the domain that would be unique or characteristic for proteins from statistically well-supported major branches of the tree.

The overall topology of the phylogenetic tree (Figure 3) is similar to that obtained for potato and *Arabidopsis *sequences (Figure 2). Most notably, a clade containing all C-type sequences of potato and *Arabidopsis*, together with additional HyPRPs from all other species studied, remains marginally statistically supported, at least in the NJ tree. Since many C-type sequences also share the characteristic long N-terminal domains and expression patterns between potato and *Arabidopsis*, we believe that, despite poor support in the ML analysis, this group may be biologically relevant. Also, the bootstrap support for the C-type clade dramatically improved (to over 70 % by both NJ and ML methods) after removal of several outlier sequences, indicating that these sequences were obviously responsible for most of the uncertainty (see Additional file 4).

Remarkably, only one HyPRP from *Pinus taeda *(Pt15) clustered outside the C-type clade. Within this clade, a branch containing nearly all remaining *Pinus taeda *sequences (except Pt1), but no angiosperm sequences, could be distinguished. The overall topology of the C-type branch suggests a possible presence of two groups of putative orthologues of St1 and St2 genes. Both groups contain sequences from all angiosperm species studied, although only one of them (the St2 group) has significant bootstrap support, at least in the NJ analysis.

To clarify the relationship of the relatively compact group of C-type sequences to the rest of the HyPRP family, we repeated the phylogenetic analysis on a representative subset of sequences from nearly all major branches. This included the *Arabidopsis thaliana *lipid transfer protein, AtLTP2 (At2g38530, [GenBank: NP_181387]), as an outgroup for rooting the tree (Additional file 4). The root of the resulting tree is located in the vicinity of the rice sequence Os03g26800, within a poorly resolved cluster of long branches outside the C-type group. This may indicate that current C-type sequences resulted either from relatively late duplications of genes encoding HyPRPs with long N-terminal domains, or from a strong selection pressure preventing diversification of these proteins. We prefer the latter explanation because of the presence of both angiosperm and gymnosperm HyPRPs within the C-type clade. All sequences with long N-terminal extensions outside the C-type cluster are highly diverged. This results in substantial erosion of the phylogenetic signal and their position remains unclear. A massive increase of bootstrap support for the C-type clade after omission of these sequences (Additional file 4) suggests that they may even be C-type HyPRPs outliers.

No statistically supported branches, including genes from all angiosperm species, were found among the remaining HyPRPs. However, several branches contain both rice and maize sequences, but only one well-supported branch harbors sequences from all dicotyledonous species (putative St10 orthologues). The majority of statistically supported branches outside C-type consist of related proteins from either a single species, or two species from the same family (either Solanaceae or Poaceae). While closely related potato and tomato HyPRPs form predominantly pairs of orthologues, the corresponding gene families in maize and rice vary substantially.

All angiosperm C-type proteins possess long proline-rich domains with high content of aliphatic amino acids, but only Pt1 and Pt11 from *Pinus taeda *have N-terminal domains of the same character. Remaining *Pinus taeda *proteins clustering in the C-type branch are shorter and rich in both proline and glycine together (formally classified as either glycine or proline-glycine rich in Figure 3). N-terminal domains of proteins outside the C-type clade are generally more variable. To our surprise, proteins with either a very short, or no N-terminal domain at all and proteins with a glycine-rich domain (instead of proline-rich one) do not form separate branches of the tree, but are more or less randomly distributed.

**Figure 5:**
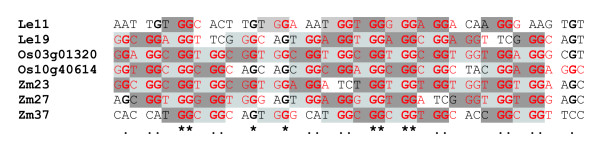
**Glycine-rich N-terminal domains may have arisen by coding sequence inversion**. An alignment of a part of the DNA sequences encoding glycine-rich N-terminal domains of several angiosperm HyPRPs (constructed manually with the aid of BioEdit) suggests a possible mechanism of independent acquisition of Gly-rich N-termini by inversion of a region encoding a part of a conventional Pro-rich domain. Triplets encoding glycine (when read left to right) are shown in red letters, while those encoding proline on the opposite strand are highlighted in gray (dark and bright shades used to distinguish individual triplets). Nucleotides conserved in at least two thirds of the sequences are shown in bold and marked by dots, absolutely conserved positions are marked by asterisks. Note the generally low degree of sequence conservation.

Analysis of nucleotide sequences encoding the highly variable N-terminal domains of several HyPRPs provides an insight into a possible mechanism of their diversification. Translation of cDNA sequences encoding the glycine-rich domains gives, in a reverse-reading frame, polypeptides rich in proline as a result of partial complementarity of codons for these two amino acids (Pro – CCX, Gly – GGX). Interestingly, cDNA sequence of Le11 translated in a reverse reading frame encodes an amino-acid motif (P-C-P-P-P-P), which can be found in proline-rich domains of some "classical" tomato HyPRPs (Figure 5). This suggests that sequences encoding the glycine-rich regions might have arisen by inversion of the corresponding part of the gene.

## Discussion

### Diversification of HyPRPs in seed plant evolution

Hybrid proline-rich proteins (HyPRPs) represent a group of secreted cell wall proteins specific to seed plants. We could find *bona fide *homologues of *HyPRP *genes only in database sequences derived from angio- and gymnosperm plants, while no representatives of this family were identified in non-seed plant species. In contrast, putative members of the lipid transfer protein (LTP) family, which share the conserved 8 CM domain with HyPRPs, were found even in a green alga (*Chlamydomonas*) and in the moss *Physcomitrella patens *(LD and LF, unpublished). We thus hypothesize that the first *HyPRP *gene may have been a *LTP *derivative that acquired a sequence encoding proline-rich N-terminal domain either by means of gene fusion or by introduction of an in-frame repetitive element. This event may have represented one of the evolutionary innovations of seed plants. This view is consistent with the previously described high flexibility and adaptability of the plant 8 CM domains (including LTPs and HyPRPs) to diverse functional requirements and sequence contexts [2].

HyPRPs exhibit high sequence diversity due to extremely variable N-terminal domains (see below) and, also, diversification of the C-terminal 8 CM domains. For instance, the C-terminal domains of two most divergent HyPRPs from potato (StHyPRP1 and StHyPRP14) share as little as 31 % of identical residues. Although frequent amino acid substitutions within the 8 CM domain often preserve the general properties of the residues at specific positions, there is apparently little, if any, selection pressure on the presence of a specific amino acid at a specific position. This results in low overall sequence conservation. Surprisingly, several HyPRPs even lacked some of the conserved eight cysteines characteristic for the 8 CM domain [2], which is believed to be stabilized by disulphide bridges formed between these cysteines [4]. However, it remains unclear whether these proteins keep the conserved spatial structure and the resulting functional characteristics of the 8 CM domain. Nevertheless, all these "mutant" HyPRPs have been found based on cDNA sequences, indicating, at least, their active transcription *in vivo*.

In spite of this variability, C-terminal domains are conserved enough to allow reliable alignment. This is a prerequisite for phylogenetic analysis (see Figure 4 and Figure 3), although the analysis is limited by the small domain size (approximately 100 residues) and poor sequence conservation. Comparison of all available HyPRPs sequences from several plant species from phylogenetically distant groups of seed plants provides insight into their evolutionary history. We have identified a specific group of HyPRPs, referred to as C-type (conserved), that contains both gymnosperm and angiosperm proteins. These proteins often share some characteristics of their N-terminal domains, namely in terms of length and amino acid composition. C-type HyPRPs exhibit less divergence than the rest of the family, suggesting that their evolution is more constrained by selection. This may be related to the ubiquitous (housekeeping) expression pattern observed at least in two dicot species. Diversification of HyPRPs evidently followed different paths in gymnosperms and angiosperms. In angiosperms, the present-day variability lies predominantly outside the C-type group, while only limited gene duplications occurred among C-type HyPRPs. However, the only gymnosperm species analyzed (*Pinus taeda*) possesses a large family of C-type proteins (the majority of them even without the long N-terminal domains), but only one HyPRP outside this group.

Orthologues shared by evolutionarily distant angiosperms (monocots and dicots) could be identified only among C-type HyPRPs. The generally weak statistical support and presence of both long and very short branches in the rest of the tree suggests an ongoing rapid diversification. Moreover, weak selection pressure apparently resulted in the accumulation of mutational changes preventing identification of ancient orthologues. The relatively recent origin of most of HyPRP diversity is also suggested by the finding that significant branches outside the C-type clade predominantly consist of multiple genes from a single species or a single family (Solanaceae or Poaceae).

*HyPRP *genes are found in clusters in both monocot (rice) and dicot (*Arabidopsis*) genomes. They are often relatively well conserved within the clusters in terms of sequence and, in case of *Arabidopsis*, even conservation of expression patterns has been observed (Figure 2). However, there is no clear relationship between genes multiplied in rice and in *Arabidopsis*, indicating that the gene clusters originated independently in the two species after their divergence. Similar evolutionary behavior has been described for the related *LTP *gene family, which also exhibits significant diversity, although no substitutions of the conserved cysteines were detected in these proteins [19]. Interestingly, at least one additional gene family containing proteins with repetitive N-terminal proline-rich domains, namely the formins (FH2 proteins), exhibits similar recent gene multiplication in *Arabidopsis *[20]. This raises the possibility that presence of repetitive sequences containing the C-C-X nucleotide motif (i.e. the proline codon) *per se *might make such genes prone to tandem duplication (although this is obviously not the only cause, as documented by the evolutionary dynamics of the LTPs).

### Multiple origins of HyPRPs with atypical N-terminal domains

BLAST searches for individual members of the HyPRP family yielded not only proteins with proline-rich N-terminal domains, but also some proteins with N-terminal domains rich in glycine (or proline and glycine). In addition, proteins consisting only of a C-terminal domain attached directly to the signal sequence, as reported previously by José-Estanyol and colleagues [2], were also found. Although the domain composition of the latter resembles non-specific lipid transfer proteins (LTPs; [21]), the C-terminal domains of these proteins cluster reliably together with the C-terminal domains of typical HyPRPs in a combined phylogenetic tree constructed from potato LTPs and C-terminal domains of HyPRPs (LD and LF, unpublished results; see also Additional file 4).

We have attempted to map the considerable diversity of the N-terminal domains onto the phylogenetic tree based on the conserved 8 CM domain. Both N-terminal and C-terminal domains of C-type proteins appear to be relatively less diversified, compared to the rest of the family. All angiosperm C-type HyPRPs, as well as two *Pinus taeda *proteins (Pt1 and Pt11), possess very long proline-rich domains rich in hydrophobic and aliphatic amino acids. This suggests that the ancestor of C-type proteins had long proline-rich domains that subsequently became shorter and were occasionally replaced by glycine-rich domains. This trend is clearly visible in *Pinus taeda*, where Pt11 with a long proline-rich domain is located at the base of the cluster of proteins, which otherwise possess shorter N-terminal domains rich in both glycine and proline (Figure 3).

In angiosperms, most of the N-terminal domain variability was detected outside the C-type clade. Glycine-rich proteins or proteins with no, or very short, N-terminal domains are more or less randomly distributed across different branches of the phylogenetic tree (Figure 3). This is in contrast to the previously published phylogeny of HyPRPs, where proteins with no, or very short, N-terminal domains were located in the central part of an unrooted phylogenetic tree calculated from whole protein sequences including the variable N-terminal domains [2]. In that study, proteins with long and short proline-rich domains formed independent branches and glycine-rich proteins defined a separate cluster within the short proline-rich domain branch. Based on these results, the authors suggested a model of HyPRP evolution involving repeat amplification or insertion of repetitive proline-rich regions into an ancestral protein lacking an N-terminal domain [2]. However, use of whole-sequence alignment for tree construction may generate artifacts in cases including significant amounts of deletions, insertions and domain rearrangements. Single-domain phylogenies are generally believed to be more reliable [22]. Based on our C-terminal domain analysis, we can deduce that HyPRPs with very short or no proline-rich domain (as well as those with glycine-rich domains) may have diverged rather recently from those with longer proline-rich domains. Since these atypical HyPRP representatives often share closely related C-terminal domains with typical HyPRPs, we believe that the loss of the proline-rich N-terminal domain or its replacement by a glycine-rich one occurred both repeatedly and independently in ancestors of different species. Thus, these "anomalous" N-terminal domains appear to be of polyphyletic origin.

High variability of N-terminal domains may be associated with their repetitive character, which is typically susceptible to rearrangements. Obvious duplication of a part of a proline-rich domain was previously documented for two putative orthologues from closely related species *Solanum brevidens *and *S*. *tuberosum *[17]. Even glycine-rich domains could, due to partial complementarity of codons for proline and glycine (Figure 5), have resulted from a rearrangement (inversion) of a sequence encoding a proline-rich domain. Since repetitive glycine- and proline- or hydroxyproline-rich proteins form the majority of cell-wall proteinaceous mass [23], this finding might have a more general evolutionary significance.

### Variability of *HyPRP *expression patterns suggests functional redundancy

For members of multigene families, analysis of gene expression patterns often provides useful clues for determining gene function. However, we could find little obvious organ specificity in the expression of the whole *HyPRP *gene family either in potato or in *Arabidopsis*. Notably, *Arabidopsis *proteins with undisputable potato orthologs (in particular those from the better-conserved C-type clade) exhibited broader organ specificity than at least some of their more divergent counterparts. This suggests that "housekeeping" HyPRPs may be subjected to more rigorous evolutionary constraints (see Figure 2). The expression patterns of individual genes were complementary, overlapping or even identical (in case of several recently amplified *Arabidopsis *genes). This suggests that expression data for a single or a few *HyPRP *genes provide little, if any, useful information on possible biological role of these proteins. Unfortunately, so far, there are practically no other "functional" data concerning the HyPRP family (reviewed in Introduction), so we can only speculate on possible functional differences on the basis of HyPRP sequence analyses.

Simultaneous expression of genes encoding HyPRPs with markedly different proline-rich domains might provide multiple modes of interactions between these proteins and the cell wall matrix. Lys-Pro motifs may interact with acidic components of the cell wall, such as pectins [24]. Serine and threonine residues, which are often present in sequences of proline-rich domains of HyPRPs, might allow hydrogen bond formation *via *their hydroxyl groups. Proline-rich domains rich in hydrophobic and aliphatic amino acids might participate in formation of hydrophobic interactions. Many *HyPRP *genes are expressed in both growing and mature organs. This suggests a possible involvement not only in the primary cell wall synthesis, but also in cell wall rearrangements in cells of mature organs, where a wide spectrum of interactions with other wall components might contribute to "fine tuning" of cell wall modifications.

However, if the observed HyPRP variability resulted from functional specialization for hypothetical interactions with other cell wall components, one would expect a relatively strong selection pressure supporting sequence conservation. This, in turn, would lead to a more robust phylogeny in respect to both the C-terminal domain tree and the distribution of variant N-terminal domains. However, this is not the case – the only stable feature of our phylogenetic tree is the presence of the apparently old group of "housekeeping" C-type HyPRPs, containing representatives of all analyzed species. While the rest of the family represents a diverse collection of highly variable sequences, C-type HyPRPs are characterized by a higher degree of sequence conservation, as well as presence of long and relatively hydrophobic N-terminal domains in all angiosperm and in two gymnosperm representatives. Interestingly, while most of the diversification of angiosperm HyPRPs (including evolution of variant, i.e. non-proline-rich, N-terminal domains) occurred outside the C-type clade, the opposite may have happened in gymnosperms, at least as far as we can judge on the basis of a single representative genome (the loblolly pine).

Sequence variability, similar to that observed for HyPRPs, was documented also for the related family of LTPs, where only the eight cysteines of the 8 CM motif were absolutely conserved [19]. Using *in vitro *assays, only loose substrate-binding specificity was found among different LTP family members; LTPs analyzed in detail bound a wide spectrum of lipidic ligands, from C10 to C18 [25,26]. Therefore, we assume that, similar to the LTPs, high variability of both C- and N- terminal domains of HyPRPs is rather a consequence of low selection pressure, possible functional redundancy, limited degree of functional specialization and high flexibility of the 8 CM domain.

## Conclusion

Hybrid proline-rich proteins (HyPRPs) could be viewed as a prototype of a dynamically evolving plant protein family constrained by rather limited structural requirements without specific demands for e.g. enzyme activity. This is consistent with the presumed role of these proteins as predominantly structural components of the plant cell walls. We have performed a detailed analysis of the *HyPRP *gene family, including both bioinformatic studies and experimental characterization of gene expression patterns, in potato (*Solanum tuberosum*). The results were compared with available data for the model plant *Arabidopsis thaliana *and several other representatives of diverse lineages of vascular plants, including two monocot species and a representative gymnosperm. We have demonstrated that the expression patterns of several genes encoding potato HyPRPs do not correlate with those of their *Arabidopsis *orthologues, pointing out the limits of extrapolation of knowledge gained by experiments in model plants towards target crop species.

Our bioinformatic study of data from seven plant species revealed that HyPRPs exhibit extraordinary variability with respect to both the sequence of their conserved 8 CM domain and overall domain structure (in particular, the length and composition of their N-terminal domains). However, we were able to reconstruct a possible evolutionary scenario that may have led to the current HyPRP diversity. We suggest that ancestral HyPRPs evolved from a lipid transfer protein (LTP) relative that had acquired a proline-rich N-terminal domain. In angiosperms, HyPRPs with long and relatively hydrophobic N-terminal domains retained (or acquired) a housekeeping expression pattern and remained relatively well conserved. We suggest terming this "conserved" clade "C-type HyPRPs". In parallel, the rest of the angiosperm *HyPRP *gene family has been undergoing continuous diversification by means of gene duplications (including tandem duplications), point mutations and rearrangements of the N-terminal domain. This resulted in repeated generation of variant (non-Pro-rich) HyPRPs with diverse expression patterns. However, the evolutionary dynamics may have been different in the gymnosperms, where diversification took place within the clade of C-type proteins. This raises the interesting possibility that in each species, the co-existence of "dynamic" and "conserved" HyPRPs might provide an evolutionary advantage. It is tempting to speculate that such a selective advantage might be related to the participation of HyPRPs in the construction of the interface between the plant cell and its environment.

## Methods

### Database searches and protein sequence analyses

HyPRP encoding sequences (including genomic, cDNA, unique transcript assemblies and unigenes) from seven plant species (*Arabidopsis thaliana*, *Lycopersicum esculentum*, *Medicago truncatula*, *Pinus taeda*, *Oryza sativa*, *Solanum tuberosum *and *Zea mays*) were identified in public species-specific sequence databases: the SOL Genomics Network [27,28] for tomato and potato; TAIR version 7 [29,30] for *Arabidopsis thaliana*; TIGR rice assembly version 5 [31,32] for rice; and the plant section of GenBank and PlantGDB [33,34] for the remaining species. Searches were performed using either TBLASTN or BLASTP [35] with default parameters and the C-terminal domain sequence of SbrPRP from *Solanum brevidens *[GenBank: U30304.2] [36] as a query. Sequences with E-values below 10^-4^, which shared the general domain organization of HyPRPs and which could be unambiguously aligned along the whole C-terminal domain without gaps inside the conserved 8 CM domain core, were considered true positives.

Utilities from the Sequence Manipulation Suite [37] were used for routine sequence handling. RADAR [38] at the European Bioinformatic Institute server [39] has been used for repeat detection and SignalP [40] for prediction of signal peptides.

Expression profiles of the *Arabidopsis *genes, including graphic representation, were obtained from the Genevestigator database [18,41].

### Sequence alignments and phylogenetic analyses

The C-terminal (non-repetitive) parts of predicted HyPRP protein sequences were aligned with the aid of ClustalX [42]. Minor manual adjustments were performed in BioEdit [43] in order to minimize the occurrence of short blocks and gaps (introduced by the sequential alignment algorithm). This also increased aligned amino acid similarity, as visually judged with the aid of a BLOSUM62-derived color code and consensus shading. Non-aligned N-ends were trimmed, leaving the master alignment presented in Figure 4 and Additional file 3. A consensus HyPRP pattern was developed on the basis of this alignment and verified by MyHits pattern search of the *Arabidopsis *GenBank proteins at the MyHits website [44,45], where it retrieved the majority of the previously known *HyPRP *genes. For the closely mutually related *Arabidopsis *and potato HyPRPs, where nearly identical amino acid sequences provided relatively little phylogenetic signal, portions of the cDNA sequence corresponding to the aligned protein sequences have been retrieved with the aid of the Sequence Manipulation Suite [37]. The alignment was then re-created manually with the aid of BioEdit's translation toggle function, using the protein sequences as a guide. The corresponding nucleotide sequence alignment was used for further analyses.

Phylogenetic trees were calculated from protein alignments after removal of all portions of the alignment where more than one sequence contained gaps longer than one residue. For nucleotide alignments, all columns containing gaps were excluded from further analysis. For tree reconstruction on the basis of protein sequences, we used either the neighbor-joining (NJ) method as implemented in the Treecon software [46] with at least 500 bootstrap samples, with Poisson correction for distance calculation, or the heuristic approximation of the maximum likelihood (ML) method [47] provided by PHYML [48] in combination with the Seqboot and Consense tools from the PHYLIP package [49,50]. In ML analyses, the JTT substitution model for amino acids or the HKY (default) model for nucleotide substitutions was used. In all cases involving either nucleotide sequences or maximum likelihood calculations, consensus NJ trees from 500 bootstrap samples were computed for comparison using PHYLIP (using the default substitution matrices, i.e. JTT for amino acids and F84 for nucleotides).

### Semiquantitative RT-PCR

Total RNA was isolated according to Stiekema and colleagues [51] from vegetative organs of potato cv. Désirée plants grown under standard *in vitro *conditions (LS medium [52], 16/8 light/dark cycle, 4 weeks). 2 μg of total RNA were used for reverse transcription with oligo-T_23 _primer and RevertAid™ M-MuLV Reverse Transcriptase (Fermentas), according to the manufacturer's instructions. Two μl of the reverse transcription reaction were used as a template for the subsequent PCR in a 50 μl reaction mixture containing 2.5 u of recombinant *Taq *DNA Polymerase (Fermentas), 1× PCR buffer with 20 mM (NH_4_)_2_SO_4 _(Fermentas), 1.5 mM MgCl_2_, 0.2 μM specific primers designed to match the part of sequence encoding the C-terminal domain (Table 1), and 0.2 mM dNTPs. The PCR was performed in a MJ Research PTC-200 cycler under the following conditions: initial denaturation (3 min, 94°C), followed by 23 cycles of denaturation (30 sec, 94°C), annealing (45 sec, 60°C) and synthesis (20 sec 72°C). As the internal standard, the transcript for the elongation factor ef1α [GenBank: AB061263] was used [53], with primers (*EF1F*: TAC TGC ACT GTG ATT GAT GCC; *EF1R*: A GCA AAT CAT TTG CTT GAC ACC; in 5' – 3'direction) newly designed to match the conserved regions of all three potato isoforms of this gene [SGN: SGN-U277726, SGN-U277730, SGN-U277731]. All samples, except those from petioles (where the amount of material was limited), were processed in parallel, starting from independent RNA isolation. As a rule, results appeared to be reproducible, both between parallels and upon repetition of the PCR step. In the rare cases, where some differences in the levels of the *HyPRP *transcript between the two parallel samples were observed, these were reproducible upon repeated PCR, even when the internal standard signal appeared constant. We believe that these differences between the two parallels were caused by variability of the starting plant material rather than by irregularities of the method (see also Results). PCR products were separated on an agarose gel in the presence of ethidium bromide and photographed in transmitted UV light using an Olympus C-4040 digital camera.

For visual comparison with the *Arabidopsis *data from the Genevestigator database in Figure 2, potato RT-PCR results were represented by a rectangular cutout from the central portion of the corresponding bands, with colors inverted using the appropriate Corel Photopaint command in order to achieve consistency with the color scheme of the Genevestigator output.

## Authors' contributions

LD carried out most of the database searches, sequence analysis and protein sequence-based phylogenetic analyses, performed the experimental study of potato *HyPRP *gene expression and contributed to writing the manuscript; FC participated in *Arabidopsis *and rice genome searches, searched and analyzed the *Arabidopsis *gene expression data, performed the nucleotide sequence-based phylogenetic analyses and participated in protein sequence analyses and in writing the paper; LF conceived of the study and its primary design, supervised the work of LD, participated in the interpretation of results, and drafted the manuscript. All authors read and approved the final manuscript.

## Supplementary Material

Additional file 1 **Cladograms of potato HyPRP protein sequences constructed**. Comparison of unrooted trees of potato HyPRP proteins constructed using the NJ and ML methods. Click here for file

Additional file 2 ***HyPRP *genes included in the study**. List of all sequences including our name, source organisms, database numbers, and chromosome cluster numbers (in case of *Arabidopsis *and rice). Click here for file

Additional file 3 **Multiple alignment of the C-terminal domains**. Colored ClustalX alignment of C-terminal domains of all HyPRPs included in the study. Minor manual adjustments were performed in BioEdit. Click here for file

Additional file 4 **A phylogenetic tree of HyPRP sequences rooted using *Arabidopsis thaliana *AtLTP2 as an outgroup**. LTP-rooted phylogenetic tree of representative sequences of all significantly supported branches from Fig. 3. The effects of the omission of three possible outlier C-type sequences on the bootstrap support of the C-type branch are indicated. Click here for file
