# Supplementary material for: Analysis of the hybrid proline-rich protein families from seven plant species suggests rapid diversification of their sequences and expression patterns
Source: BMC Genomics. 2007 Nov 12;8:412. doi: 10.1186/1471-2164-8-412 (PMC2216038; doi:10.1186/1471-2164-8-412)
Supplement: Additional file 1 — Cladograms of potato HyPRP protein sequences constructed. Comparison of unrooted trees of potato HyPRP proteins constructed using the NJ and ML methods. [file 1471-2164-8-412-S1.pdf]

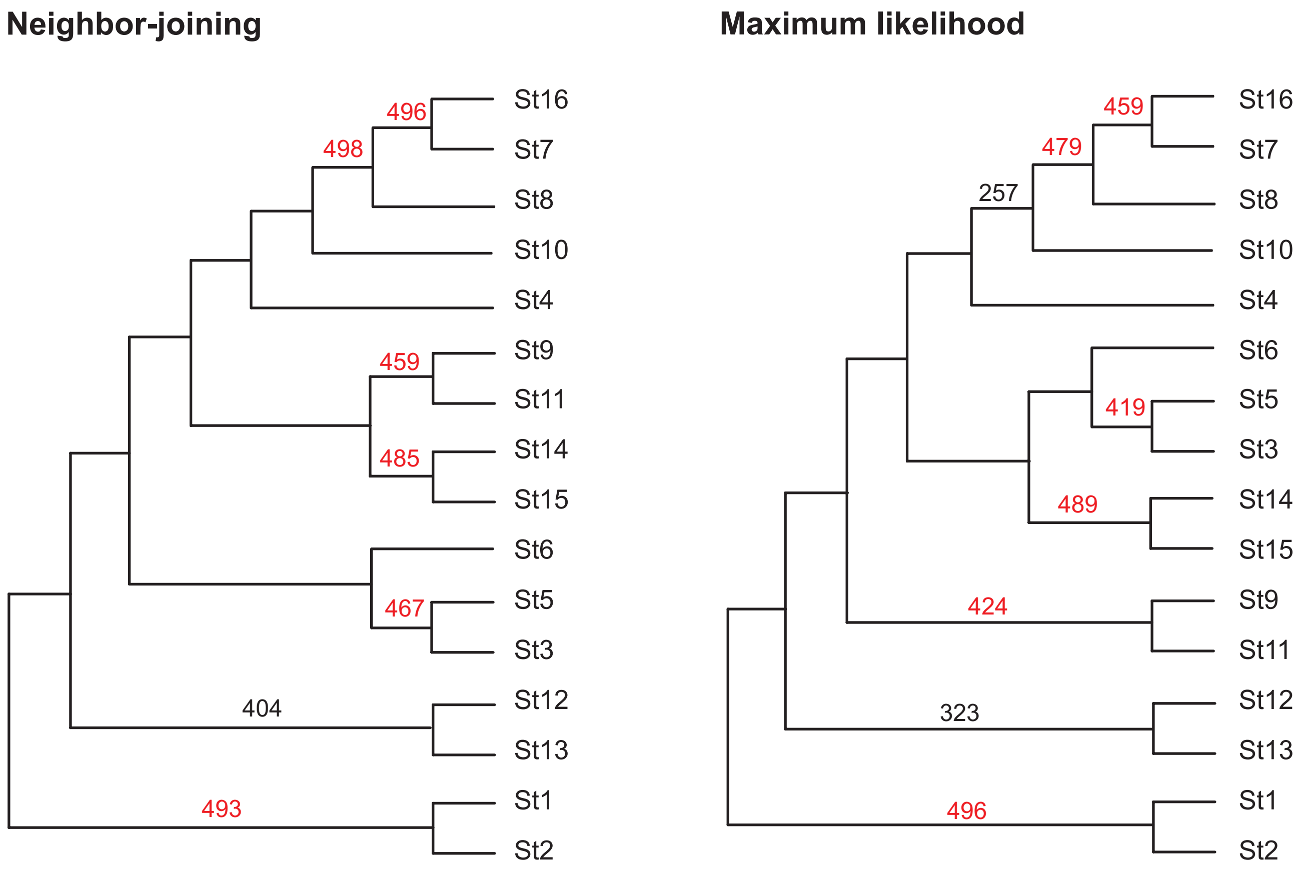

Cladograms of potato HyPRP protein sequences constructed using the NJ and ML methods. Both trees are unrooted, bootstrap values above 50 % (from 500 replicates) are shown above branches. Bootstrap values in red denote branches that were supported by both methods and also recovered in trees based on nucleotide sequences (see Figure 1).
