# Supplementary material for: Analysis of the hybrid proline-rich protein families from seven plant species suggests rapid diversification of their sequences and expression patterns
Source: BMC Genomics. 2007 Nov 12;8:412. doi: 10.1186/1471-2164-8-412 (PMC2216038; doi:10.1186/1471-2164-8-412)
Supplement: Additional file 3 — Multiple alignment of the C-terminal domains. Colored ClustalX alignment of C-terminal domains of all HyPRPs included in the study. Minor manual adjustments were performed in BioEdit. [file 1471-2164-8-412-S3.pdf]

```
                    10        20        30        40        50        60        70        80        90       100       110       120
           ....|....|....|....|....|....|....|....|....|....|....|....|....|....|....|....|....|....|....|....|....|....|....|....|....|
At4g12490  SSGNCPIDA-LRLGVCANVL------SGLLNVQLGQPSP------QPCCSLIQGLVDLDAA-VCLCTALRANVLGI-NLNVPISLSVLL--NVCNRRLP--SNFQCA------------------
At4g12500  SSGNCPIDA-LRLGVCANVL------SGLLNVQLGQPSA------QPCCSLIQGLVDLDAA-ICLCTALRANVLGI-NLNVPISLSVLL--NVCNRRLP--SDFQCA------------------
At4g12480  SSGNCPIDA-LRLGVCANVL------SSLLNIQLGQPSA------QPCCSLIQGLVDLDAA-ICLCTALRANVLGI-NLNVPISLSVLL--NVCNRKVP--SGFQCA------------------
At4g12470  SGNSCPIDA-LKLGVCANVL------SSLLNIQLGQPSS------QQCCSLIQGLVDVDAA-ICLCTALRANVLGI-NLNVPISLSVLL--NVCNRKLP--SGFQCA------------------
At1g62510  AIAKCPRDA-LKLGVCANVL------NGLLNVTLGKPPV------EPCCTLIQGLADLEAA-ACLCTALKANILGI-NLNIPLSLSLLL--NVCSKKVP--RGFQC-------------------
At1g12090  GSSKCPKDT-LKLGVCANVL------NGLLDLTLGKPPV------EPCCSLIQGLADVEAA-VCLCTALKANILGI-NLNLPISLSLLL--NVCSKQLP--PGFQC-------------------
At4g12520  STGSCPKDT-LKLGVCANVL------KDLLKIQLGTPPV------KPCCSLLNGLVDLEAA-ACLCTALKAKVLGI-NLNVPVSLSLLL--NVCGKKVP--SGFVCA------------------
At4g12510  STGSCPKDT-LKLGVCANVL------KDLLKIQLGTPPV------KPCCSLLNGLVDLEAA-ACLCTALKAKVLGI-NLNVPVSLSLLL--NVCGKKVP--SGFVCA------------------
At2g45180  VKPTCPTDT-LKLGVCADLL-------GLVNVVVGSPPK------TPCCTLLQGLANLEAA-VCLCTALKANVLGI-NLNVPIDLTLLL--NYCGKKVP--HGFQCS------------------
At4g12550  TTGTCPKNS-IEIGTCVTVL-------NLVDLTLGNPPV------KPCCSLIQGLADLEAA-VCLCTAVKASILGIVNINLPINLSVLL--NVCSRNAP--KSFQCA------------------
At4g22460  TTGTCPKDA-LKVGVCVNAL------NLLNGLTPGTPPV------TPCCSLIEGLVDLEAA-ICLCTALKASVLGI-NLTLPINLSLLL--NICNREAS--RDFQFP------------------
At4g12545  TTGTCPIQ----ISTCANVL-------NLVDLTLGNPPV------KPCCSLIQGLADLEAA-ACLCTALKASILGIVNINLPINLSVLL--NVCSRNAP--KGFQCA------------------
At4g12530  ADNTCPRDV-LKLSTCSNVL-------NLINLKLGAPAM------RPCCSILFGLIDLDVA-VCLCTALKLSLLGI-TIDTPIHLNLAL--NACGGTLP--DGFRCPT-----------------
At1g62500  KQPTCPINA-LKLGACVDVL------GGLIHIGLGNPVE------NVCCPVLQGLLELEAA-VCLCTTIRLKLLNL-NIFIPLALQALI---TCGINPP--SGFVCPPLT---------------
At2g10940  GKATCPIDT-LKLGACVDLL------GGLVKIGLGDPAV------NKCCPLLKGLVEVEAA-ACLCTTLKLKALDL-NLYVPVALQLLL---TCGKNPP--PGYTCSI-----------------
At4g00165  ATTKCPRDT-LKFGVCGSWL------GLVSEV-IGTPPS------QECCSLIKGLADFEAA-VCLCTALKTSILGVAPVKIPVALTLLL--NSCGKNVP--QGFVC-------------------
At3g22120  IPETCPIDT-LKLGACVDVL------GGLIHIGLGKSHAK-----AECCPVLGGLLDLDAA-VCLCTTIKLKLLNI-DLVLPIALELLL---DCGKTPP--SDFKCPA-----------------
At4g15160  KPETCPIDA-LKLGACVDVL------GGLIHIGLGKSYAK-----AKCCPLLDDLVGLDAA-VCLCTTIRAKLLNI-DLIIPIALEVLV---DCGKTPPP-RGFKCPTPLKRTPLL---------
At4g22490  NFGSCPRNP-LQLGVCANVL-------GLANVTAGDPRA------RQCCTALNGLTNVQVT-DCLCFIFRPIPLVF-GIDV-AVREIFF---ACNRVFP--IGFQCPPPQ---------------
At4g22520  QAPVCPRDS-TQFLSCTNVL-------NLSLILINNQSV------LPCCTLVTGLDAATAS-ACICNAVRITIFNF-LTINVRVNQVLR---LCRIIPP--LGFRCP------------------
At3g22142  MPKTCPIDT-LKLGSCVDLL------GGLVHIGIGKSAK------EKCCPVVEGLVDLDAA-VCLCTTIKAKLLNI-DVILPIALEVLL---NCGKNPP--PGFKCPA-----------------
At4g22517  AQPVCPLNR-SDLGICVNVL-------GLISLTTSNVA--------RCCSILAGLNNTPLVSVCACEAVRLNILNLLGITVNLNLRLNGLLGLCRLPVQ--PGFNCLL-----------------
At4g22485  PRGTCPRNA-NQIRTCSNVL------RFFGNFLDFRLA-------QPCCTLIRNLSDAEAA-ACLCDLVRARRS----TLSPNIIILCR---ACGRRIP--RGFTCP------------------
At1g12100  TTPSCSRDA-IKLGVCAKIL------DVAVGTVIGNPSD------TLCCSVLQGLVDLDAA-VCLCTTIKANILGI-NIDLPISLSLLI--NTCGKKLP--SDCICA------------------
At4g22610  QPPTCPRDI----EACSNVF-------GLGRILNYQTV-------RPCCTLVDGLDAYVAS-VCICDSIKIYGISY--PFTVGLVQVLS---LCGADYPP-PGFRCRENEISVLSYNNGASMAL*
At5g46900  PKPTC-KDA-LKLKVCANVL-------DLVKVSLPPTS--------NCCALIKGLVDLEAA-VCLCTALKANVLGI-NLNVPISLNVVL--NHCGKKVP--SGFKCA------------------
At5g46890  LKPTC-KDA-LKLKVCANVL-------DVVKVSLPPTS--------NCCALIKGLVDLEAA-VCLCTALKANVLGI-NLNVPISLNVVL--NHCGKKVP--SGFKCA------------------
At4g22470  LVGICSKND-TELKICAGIL-------AISDGLLTTGRA------EPCCSIVRNVSDLDAV-TCFCKSVGARRF----SLSPNFGIFFK---VCGRRIP--QGFSCP------------------
St1        AQPTCPIDA-LKLGACVDVL------GGLIHIGIGGSAK------QTCCPLLGGLVDLDAA-ICLCTTIRLKLLNI-NIILPIALQVLI--DDCGKYPP--KDFKCPST----------------
St2        TKATCPIDT-LKLGACVDLL------GGLVHIGLGDPAV------NECCPILSGLVELEAA-ACLCTTLKVKLLNL-KIYVPLALQLLV--TCGKSPPP---GYTCSI-----------------
St3        SKGKCPKDT-LKLNACANLL------NDLVHLVIGSSPAK-----TKCCSLIHGLADLDAA-VCLCTALKANVLGI-NLNVPLSLSLLL--NNCGKYVP--KDFQCA------------------
St4        SKGKCPIDT-LKLGVCANVL------GNLLGVVLGNPPK------KPCCSLIEGLVDLEAA-LCLCTAIKANILGI-NLNVPLSLSLLL--NVCGKKAP--SGFQCPK-----------------
St5        SKGTCLKDT-LKLNACANLL------GDLLHLVIGSSPTK-----TKCCSLIEGLVDLDAA-VCLCTALKANLLGI-NLDIPLSLNLLL--NNCGKYAP--KNFQCA------------------
St6        TYHKCPKDT-LKLKVCANLL------NDLVHVVIGSPLLSS---KSSCCSLIENLADVDAA-VCLCTAIKANVLGA-HLNAALSLSLLL--NNCGKTAP--KGFKCA------------------
St7        KEDKCPKDA-LKLGVCANVL------SGLLNVTLGTPPV------KPCCSLIGNLVDLEAA-VCLCTALKANILGI-NLNIPISLSLLL--NVCSKDVP--KGFICA------------------
St8        SQTKCPKDA-LKLSICANVL------NGLLNVTLGTPPV------KPCCSLIGNLVDLEAA-DCLCTALKANILGI-NLNIPISLSLLL--NVCSKEAP--KEFICP------------------
St9        KYKTCPIDT-LKLGVCADVL-------GLVNVVVGSPPV------TPCCSLISGLADVEAA-LCLCTALKANVLGI-NLNVPISLSLLL--NVCSKKVP--YGFQCPN-----------------
St10       SKGHCPRDA-LKLGVCANVL------NGPIGAVIGTPPD------PHCCMVLGGLLDLEAA-ICLCTALKANILGI-NINIPIALSLLI--NTCGKTLP--SDFICA------------------
St11       PKETCPIDT-LKLGVCADVL-------GLVNVIVGSPPV------TPCCSLLSGLANAEAA-ICLCTALKANILGI-NLNLPISLSLLL--NVCSKEAP--AGFQCS------------------
St12       APSKCPKDT-LKFGICGDWL-------GLVHEVIGAKPS------SKCCALLEGIADVEAA-LCLCTAINANVLGVLKLKVPIAISVVL--NSCGKKVP--KGFKCA------------------
St13       VNPYCPRDT-LKLGVCGDLL-------GLVNVAIGSQVT------TPCCSLLEGLADLEVA-ACLCTAIKANVLGIIKLDIPVALSALV--SACAKKVP--TGFKCG------------------
St14       GQQTCP--T-LRLGVCASIL-------NVVNVTVGSPPT------MPCCSLIQGLTDLEAA-ACLCAAIRANVLGIITLNPTVPINLIL--SACGRSLP--ADFTC-------------------
St16       KEGKCPKDA-LKLGVCANVL------SGLLNVTLGTPPV------KPCCSLIGNLVDLEAA-VCLCTALKANILGI-NLNIPISLSLLL--NVCSKDVP--KGFICA------------------
St15       GQVRCPRDA-LKLGVCANIL-------NLVNVVVGSPPT------LPCCSLIQGLANLEVA-ACLCTAIRANILGL-NLNVPLTLSLIL--NNCGMNNS---GFTC-------------------
Pt1        PATTCPLDT-LKLGACVDVL------GGLVHVGIGDPVV------NQCCPVLQGVLALEAA-VCLCTTIKASVLNL-NIVLPLALELLV---ACGKSPP--SGFTCP------------------
Pt2        GGGSCPIDA-LKLGACVDLL------GGLVHVGLGDPTV------NQCCPLIQGVLELEAA-LCFCTTIRLKLLNL-NIILPLALELFV---QCGTTPP--PGFKCPPLN---------------
Pt3        GGSSCPLDA-LKLGACVDLL------GGLVHIGIGDPVV------DQCCPLIQGVAALEAA-LCLCTTIRAKLLNL-NVILPLALELFV---QCGVTPP--PGFTCPSL----------------
Pt4        GSSSCPLDA-LKLGACVDLL------GGLVHVGIGDPVV------NQCCPVLAGVLELEAA-LCLCTTIRLKLLNL-NVILPLALELFV---QCGLAPP--PGFTCPPLN---------------
Pt5        GSSSCPLDA-LKLGACVDLL------GGLVHVGIGDPVV------NQCCPVLAGVLELEAA-LCLCTTIRLKLLNL-NVILPLALELFV---QCGLTPP--PGFTCPPLN---------------
Pt6        GSSSCPLDA-LKLGACVDLL------GGLVHVGIGDPVV------NKCCPVLAGVLELEAA-LCLCTTIRLKLLNL-NVILPLALELFV---QCGLTPP--PGFTCPPLN---------------
Pt7        GSSSCPLDA-LKLGACVDLL------GGLVHVGIGDPVV------NQCCPVLAGVLELEAA-LCLCTTIRVKLLNL-NVILPLALGLFV---QCGLTPP--PGFTCPPLN---------------
Pt8        STAKCPLNA-LKLGACVDLL------QGLVHVGLGDPVV------NQCCPLIQGVAALEAA-LCLCTTIRAKVLSL-NVLLPIALSLVA---SCGLTVP--PDFKCPAN----------------
Pt9        GSSSCPLDA-LKLGACVDLL------GSLVHLGIGDPVV------NQCCPVIQGVLELEAA-LCLCTTIRLKLLNL-NVILPLALELFA---QCGLTPP--PGFTCPPLN---------------
Pt10       SFSSCPLDA-LKLGACVDLL------GSLVHLGVGDPVV------NQCCPVIQGVLELEAA-LCFCTTIRLKLLNL-NVILPLALELFV---QCGLTPP--PGFTCPPLN---------------
Pt11       SNPKCPKDT-LKLGACVDLL------GGLVHVIIGDPVV------NECCPVIQGLAGLEAA-LCLCTAIRAKVLSL-NVYLPLALELIA---SCGLTPP--EGFKCPEAS---------------
Pt12       XTAKCPLNX-LKLGACVDLX------QGLVHVGLGDPVV------NQCCPLIQGVAALEAA-LCLCTTIRAKVLNL-NVLLPIALSLVA---SCGLTVP--PDFKCPAN----------------
```

```
Pt13   GGSSCPLDA-LKLGACVDLL------EGLVHVVIGDPVV------NQCCPLIEGVAALEAA-LCLCTTIRLKLLDV-NVILPLALELFV---QCGLTPP--PGFTCPPL----------------
Pt14   GSSSCPLDA-LKLGACVDLL------GSLVNLGIGDPDV------NQCCPVIQGVLELEAA-LCFCTTIRLKLLNL-NVILPLALELFV---QCGVTPP--PGFTCPPLN---------------
Pt15   PQSSCPLANPVSLNVCVDLL-------GLVHVVLGNPNT------AECCDIING-LGVDAT-VCLCTAIHLKVLGL-NVDIPLPLKLLV---SCGQDLP--NGLTC-------------------
Pt16   STPKCPLNA-LKLGACVDLL------QGLVHVGLGDPVV------NQCCPLIQGVAALEAA-LCLCTTIRAKVLSL-NVLLPIALSLVA---SCGLTVP--PDFKCPAN----------------
Pt17   GSSSCPLDA-LKLGACVDLL------GGLVHVGIGDPVV------NQCCPLIQGVAALEAA-LCLCTTIRAKLLNL-NVILPLALELFV---QCGVTPP--PGFTCPTL----------------
Pt18   GSSSCPLDA-LKLGAFVDLL------GGLVHVGIGDPVV------NQCCPVLAGVLELEAA-LCLCTTIRLKLLNL-NVILPLALELFV---QCGFTPP--PGFTCPPLS---------------
Pt19   STAKCPLNA-LKLGACVDLL------QGLVHVGLGDPVV------NQCCPLIQGVAALEXX-LCLCTTXXAXXLXL-NVLLPXALSLVA---SCGLTVP--PNFKCPAN----------------
Pt20   SNPKCPKDT-LKLGACVDLL------XGLVHVLLFKKIX------XXCWGGKHNLAGLEAA-LCLCTAIRKKILSL-NVYLPLALELIA---SCGLTPP--EGFKCPEAS---------------
Pt21   GSTSCPLDA-LKLGACVDLL------GGLVHVGIGDPVV------NQCCPVLAGVLELEAA-LCLCTTIRLKLLNL-NVILPLALELFV---QCGLTPP--PGFTCPPLN---------------
Zm2    PTGKCPVDT-LKLLACVDAL------NGLVHAVVGTNAS------DTCCPLLSGVADLDAA-LCLCTTIKAKALSV-SLVLPVAISVLV--NECGKHVP--SSFQCP------------------
Zm4    PTGKCPVNT-LKLLACVDAL------NGLVHAVIGTKAS------DTCCPLLSGVADLDAA-LCLCTTIKAKALSV-SLVLPVAISVLV--NECGKHVP--SSFQCP------------------
Zm14   PRGKCPVNT-LKLLACVDAL------NGFVHAVFGTKAR------DTCCPLFSGVADLDAA-FCFCPPIKAKAFRV-SLVFPVAISVFV--NEWGKHVP--SSFQCP------------------
Zm16   RRGKCPVNT-LKLLAWVDAL------NGFVHAVFGTKAS------DTCCPLFSGVADLDAA-FCFCTTIKAKGFRV-SFVFPVAISVFV--NEWGKHVP--SSFQCP------------------
Zm6    AVRTCPIDT-LKLNACVDVL------SGLIHLVIGQEAR------SKCCPLVQGVADLDAA-LCLCTTIRARLLNI-NIYLPIALNLLI---TCGKHAP--SGFQCPPLYD--------------
Zm34   AGGTCPIDT-LKLNAWGDVL------SGLIPLVIGQEAR------SKCCPLVQGGGDLDAA-LCLCTPIRGGLLNI-NIYLPIALNLVI---TWGKHAP--SGFQCPPLFD--------------
Zm3    SSPTCPADS-LKLGACVDLL------GGLVHIGLGDPVV------NKCCPVLEGLVELEAA-VCLCTTIKLRLLNI-NLYLPLALQLLL---TCGKTPP--PGYTCTV-----------------
Zm5    SSPTCPADS-LKLGACVDLL------GGLVHIGLGDPVV------NKCCPVLEGLVELEAA-ECLCTTIKLRLLNI-NLYLPLALQLLL---TCGKTPP--PGYTCTV-----------------
Zm1    SSPTCPADS-LKLGACVDLL------GGLVHIGLGDPVV------NKCCPVLEGLVELEAA-VCLCTTIKLQLLNI-NLFLPLALQLLL---TCGKTPP--PGFTCPV-----------------
Zm12   SHGRCPIDA-LKLKVCANVL---------GLVKVGLPQH------EQCCPLLEGLVDLDAA-LCLCTAIKANVLGI-HLNVPLSLNLIL--NNCGKICP--EDFTCPN-----------------
Zm18   SHGRCPIDA-LKLKVCANVL---------GLVKVGLPQH------EQCCPLLEGLVDLDVA-LCLCTAIKANVLGI-HLNVPLSLNLIL--NNCGKICP--EDFTCPN-----------------
Zm9    SHGRCPIDA-LKLKVCANVL---------GLVNVGLPQH------EQCCPLLEGLVDLDAA-LCLCTAIKANVLGI-HLNVPLSLNLIL--NNCGKICP--EDFTCPN-----------------
Zm35   SHGRCPMDA-FKLKVCANVL---------GFVKVGLPQH------EQCCPLLEGLVDLDAA-LCFCTAIKANVLGI-HLNVPFSFNFIL--NNWGKICP--EDFTCPN-----------------
Zm13   SHGRCPIDA-LKLKVCANVL---------GLVKVGLPQY------EQCCPLLEGLVDLDAA-LCLCTAIKANVLGI-HLNVPLSLNLIL--NNCGKICP--EDFTCPN-----------------
Zm17   SHGRCPIDA-LKLKVCANVL---------GLVKVGLPQY------EQCCPLLEGLVDLDAA-LCLCTAIKANVLGI-HLNVPLSLNLIL--NNCGRICP--EDFTCPN-----------------
Zm10   SHGRCPIDA-LKLKVCANVL---------GLVKVGLPQP------EQCCPLLEGLVDLDAA-LCLCTAIKANVLGI-HLNVPLSLNLIL--NNCGKICP--EDFTCPN-----------------
Zm7    SHGRCPIDA-LKLKVCANVL---------GLVKVGLPQH------EQCCPLLEGLVDLDAA-LCLCTAIKANVLDI-HLNVPLSLNLIL--NNCGKICP--KDFTCPN-----------------
Zm8    SHGRCPIDA-LKLKVCANVL---------GLVKVGLPQH------EQCCPLLEGLVDLDAA-LCLCTAIKANVLGI-HLNVPLSLNLIL--NNCGKICP--KDFTCPN-----------------
Zm11   SHGRCPIDT-LKLKVCANVL---------GLVKVGLPQH------EQCCPLLEGLVDLDAA-LCLCTAIKANVLGI-DLNVPLSLNLIL--NNCGKICP--KDFTCPN-----------------
Zm15   SHGRCPIDA-LKLKVCANVL---------DLVKVGLPQH------EQCCPLLEGLVDLDAA-LCLCTAIKANVLGI-HLNVPLSLNLIL--NNCGKICP--KDFTCPN-----------------
Zm19   SHGRCPIDA-LKLKVCANVL---------GLVKVGLPQY------EQCCPLLEGLEDLDAA-LCLCTVIKANALGI-HLNAPLSLNLIL--NNCSKICP--EDFTCPN-----------------
Zm33   SAGRCPINA-LKLKGCANVL---------DLVKGGLPQH------EQCCPVLEGLVDLDAG-LCLCTGIKANGLGI-HLNGPLSLNLIL--NNCGKICP--KDFTCPN-----------------
Zm45   TTGACPINA-LKLKVGANGL---------DLGKGGLPQH------EQCCPLLEGLVDLDAA-LGLCTGIKANGLGI-HLNGPLSLNLIL--NNCGKICP--KDFTCPN-----------------
Zm44   SAGSCPIDA-LKLEVCANVL-------NLLRLNIGVPDD------EQCCPLLQGLADLDAA-VCLCLAIRANILGI-VLNVPIDLTLLL--NYCHKDRV--ASFTCPA-----------------
Zm52   GGNPCPTSAVADLKVCADVL-------VLLKLRINVPQG------QQCCPLLGGLLDLDLA-ACLCAAIRLNALGV-PVNLPLDVPLVL--NYCGRNASAVPGSNCPF-----------------
Zm29   GHGSCPRNA-LKLGVCANVL-------GLVKAKVGSPPY------EPCCSLLDGLVDLEAA-VCLCTAVKANILGI-NLNLPIDLSLIL--NNCGKNCP--NDFHCA------------------
Zm20   -FGKCPRDA-LKMGVCANVL-------GLIKAKVGVPPT------EPCCPLLKGLVDLEAA-VCLCTAIKGEVLGI-KLNLPVDLSLIL--NHCGKTVP--TGFKCL------------------
Zm22   SFGKCPRDA-LKLGVCANVL-------GLIKAKVGVPPT------EPCCPLLKGLVDLEAA-VCLCTAIKGEVLGI-KLNLPVDLSLIL--NHCGKTVP--TGFKCL------------------
Zm21   SFGKCPRDA-LKLGVCANVL-------GLIKAKVGVPPT------EPCCPLLKGLVDLEAA-VCLCTAIKGEVLGI-KLNLPVDLSLIL--NHCGKRVP--TGFKCL------------------
Zm30   SFGKCPRDA-LKLGVCANVL-------GLIKAKVGVPPT------EPCCRLLEGLVDLEAA-VCLCTAIKGEVLGI-KLNLPVDLSLIL--NHCGKTVP--TGFKCL------------------
Zm26   SFGRCPRDA-LKLGVCANVL-------GLIKAKVGVPPA------EPCCPLLEGLVDLEAA-VCLCTAIKGQILGI-NLNLPIDLSLIL--NYCGRTVP--TGFKC-------------------
Zm43   SFGRCPRDA-FKLGVCANVL-------GLIKAKVGVPPE------EPSCPLLEGVVDLEAT-VCLCTSIKGQILGI-NLNLPIDLSLIL--KYCGRTVP--TGFKC-------------------
Zm28   SFGRCPRDA-LKLGVCANVL-------GLIKAKVGAPPA------EPCCPLLEGLVDLEAA-ACLCTAIKGNILGI-NLNLPVDLSLIL--NYCGRTVP--TGFKC-------------------
Zm37   WYGHCPTNA-LKLGVCANVL-------DLIKAKVGVPVN------EPCCPLLNGLVELEAA-VCLCTAIKANVLGL-NLNIPVNLSLVL--NFCGKGVP--TGFKCL------------------
Zm23   TSGRCPVDA-LKLGVCANLL------NGLINATLGTPPR------TPCCTLIQGLADLEAA-VCLCTVLRANVLGI-NLNLPINLSLLV--NYCGRRVP--SGFQCF------------------
Zm27   TSGRCPVDA-LKLGVCANLL------NGLINATLGTPPR------TPCCTLIQGLADLEGA-VCLCTVLRANVLGI-NLNLPINLSLLV--NYCGRGVP--SGFQCF------------------
Zm39   ---RCTVDA-LKLGVCANML------NGLINATLGTPLR------TPWCTLIQGLADLEAA-LCLCTVLRANVVGI-NLNLPINLSLLV--NYCGRRVP--SGFQCF------------------
Zm36   GKGKCPKDT-LKLGVCANVL------SGLLDLTLGKPPV------EPCCSLIQGLADVEAA-VCLCTALKANVLGI-NLNLPISLSLLL--NVCSKQVP--PGFQC-------------------
Zm31   SGGKCPKNA-LKLGVCANVL-------GLVKVSIGKVPT------DSCCPLLDGLADLEAA-VCLCTALKANVLGI-NLDVPVKLTLLL--NYCGKSVP--QGFLCA------------------
Zm32   SGGKCPKNA-LKLGVCANVL-------GLVKVSIGKVPT------DSCCPLLDGLADLEAA-VCLCTALKANVLGV-NLDVPVKLTLLL--NYCGKSVP--QGFLCA------------------
Zm41   SSGKCPLNA-LKFGVCADVL-------GLVKGEAGKVPA------EPCCTLIKGLADFEAA-VCLCTAIKANVLGV-VIDVPIKLSALV--NYCGKCVP--KGYLCA------------------
Zm48   SNGKCPLNA-LKFGVCADVL-------GLVKGEAGKVPA------EPCCTLIKGLADFEAA-VCLCTAIKANVLGV-VIDVPIKLSALV--NYCGKCVH--EKYLCA------------------
Zm38   SSGKCPLNA-LKFGVCADVL-------GLVKGEVGKVPA------EPCCTLIKGLADFEAA-VCLCTAIKANVLGV-VVDVPIKLSALV--NYCGKCVP--KGYMCA------------------
Zm46   SSGKCPLNA-LKFGVCVNAL-------GLVKGEAGKVPA------EPCCNLIKGLADFEAA-VCLCTAIKGNVLGV-VFDVPIKFNALV--NYCGKCVP--KGYMCA------------------
Zm50   FSGKCPLNA-LKFGVWVNAL-------GLVKGEAGKVPA------EPCCNLIKGLADLEAA-VCFCTAIKGKVLGV-VFDVPIKFNALV--NYCGKCVP--KGYMCG------------------
Zm47   TGGKCPKHA-LKFAACANVL-------GLVSAEVGHPPA------EPCCSILGGLADLEAA-VCLCTAIKANVLGI-TVDIPVKLSLII--NYCGKNLP--SGFICA------------------
Zm24   SSDKCPIDA-LKLGACVDVL--------GNEVHVGDANV-------QCCPLVKGIAGLSAA-ACLCTAIKAKVLDI-SVYVPIALEVLV---NCGCAVP--PGYKCT------------------
Zm25   SSDKCPIDA-LKLGACVDIL--------GNEVHIGDANV-------KCCPLVKGIAGLSAA-ACLCTAIKAKVLDI-SVYVPIALEVLV---NCGCEVP--PGYKCT------------------
Zm42   SLDRCPIDA-LKLGACVDVF--------GNEVHVGDANV-------QCCPLVKGIAGLFAA-GGLCTAIKAKVLDI-SVYVPIALEVLV---NCGWAVP--PGYKCT------------------
```

```
Zm49        SLDKCPIDA-LKLGACVDVF--------GNEVHVGDANV-------QCCPFVKGIAGLFGG-GCLCTAIKAKVLDI-SVYVPIALEVFV---NWGWAVP--PGYKCT------------------
Zm40        SWDKCPIDA-LKLGACVAIF--------GNEVHIGDANV-------KCCPLVKGIAGLSAA-GCLCTPIKAKVRDI-SVYVPIALGVLV---NCGWEVP--PGYKCT------------------
Zm51        TRLFCPWDA-VKFGACVGLL--------GAAGLQGGAQLG-----SKCCDVVQGLAAAEVG-ACFCTTVKEAVLGV-PTEWDVGVGVLA--SACKTELP--DGFKCV------------------
Le11        GQGKCPKDA-LKLGVCANVL-------NLVNVTVGSPPP------LPCCSLIQGLTDLEAA-VCLCTAIRANVLGI-NLNIPLSLSLVL--NNCGRNPP--TGFTC-------------------
Le1         AQPTCPIDA-LKLGACVDVL------GGLIHIGIGGSAK------QTCCPLLGGLVDLDAA-ICLCTTIRLKLLNI-NIILPIALQVLI--DDCGKYPP--KDFKCPST----------------
Le2         TKATCPIDT-LKLGACVDLL------GGLVHIGLGDPAV------NECCPILSGLVELEAA-ACLCTTLKVKLLNL-KIYVPLALQLLV---TCGKSPP--PGYTCSI-----------------
Le5         SKGKCPIDT-LKLGVCANVL------GNLLGVVLGNPPK------KPCCSLIEGLVDLEAA-LCLCTAIKANILGI-NLNVPLSLSLLL--NVCGKKAP--SGFQCPK-----------------
Le6         SQTKCPRDA-LKLGVCANVL------NGLLNVTLGTPPV------KPCCSLLGNLVDLEAA-VCLCTALKANILGI-NLDIPISLSLLL--NVCTKEAP--KGFICS------------------
Le7         KEGKCPKDT-LKLGVCANVL------SGLLNVTLGTPPV------KPCCSLIGNLVDLEAA-ACLCTALKANVLGI-NLNIPISLSLLL--NVCSKDVP--KGFICA------------------
Le8         KYKTCPIDT-LKLGVCANVL-------GLVNVVVGSPPV------TPCCSLISGLADVEAA-LCLCTALKANVLGI-NLNVPISLSLLL--NVCSKKVP--NGFQCPN-----------------
Le9         SKGHCPRDA-LKLGVCANVL------NGPVGAVIGTPPD------PHCCMVLGGLLDLEAA-VCLCTALKANILGI-NINIPIALSLLI--NTCGKTLP--SDFICA------------------
Le10        VSADCSTDI-LKFGACTNIL------NDLVGVIIGTTPT------SSCCSLIDGLLDLEAA-VCLCTALKANVLGI-NLDIPLSLNILL--NVCGKKYP--TGYTC-------------------
Le12        VSADCSTDI-LKFGACTNIL------NDLVGVIIGTTPT------SSCCSLIGGLVDLEAA-VCLCTAIKADILGI-HLDIPISLNILL--NVCGKNYP--TGYTC-------------------
Le13        PKETCPIDT-LKLGVCADVL-------GLVNVVVGSPPV------TPCCTLLSGLANAEAA-LCLCTALKANILGI-NLNLPISLSLLL--NVCSKEAP--AGFQCS------------------
Le14        SQQTCSIDT-LKLGVCANIL-------NLVNVIVGSPPT------LPCCSLIQGLTDLEAA-VCLCTAIKANVLGI-NLNVPLSLSLIL--NTCGKKYP--TGFTC-------------------
Le15        VNPYCPRDT-LKLGVCADLL-------GLVNVAIGSQVT------TPCCSLLEGLADLEVA-ACLCTAIKANVLGI-VKLDIPVALSALV-SACAKKVPT-GFKCG-------------------
Le16        PPATCPIDA-LKLGLCLDVL------GGLVHIGIGNPVE------HICCPVLQGLLELEAA-ICLCTTIRLKLLNL-NIFLPLALSVLA---TCGLTPP--PGFVCPPLV---------------
Le19        GQVRCPRDA-LKLGVCANVL-------NLVNVVVGSPPT------LPCCSLIQGLANLEVA-ACLCTAIRANILGL-NLNVPLTLSLIL--NNCGMNN---SGFTC-------------------
Le3         SKGKCPKDT-LKLNACANLL------GDLLHLVVGSSPA-----KTQCCSLIEGLVDLDAA-VCLCTALKANVLGI-NLNIPLSLSLLL--NNCGKYAP--KNFQCK------------------
Le4         SKGKCPKDT-LKLNACANLL------NDLVHLVIGSSPA-----KTKCCSLIHGLADLDAA-VCLCTALKANLLGI-NLNVPLSLSLLL--NNCGKYVP--KDFQC-------------------
Le17        SKGKCPKDT-LKLNACANLL------GDLLHLVVGSSPA-----KTQCCSLIEGLVDLDAA-VCLCTALKANVLGI-NLNVPLSLSLLL--NNCGKYAP--KNFQCA------------------
Le18        TYHKCPKNT-LKLKVCANLL------NDLVHVVVGSPLLSS---KSSCCSLIENLADVDAA-VCLCTAIKANVLGA-HLNAALSLSLLL--NNCGKTAP--KGFKCA------------------
Mt10        AQQTCSIDA-LKLGACVDVL------GGLIHIGIGGSAK------QTCCPLLQGLVDLDAA-ICLCTTIRLKLLNI-NLVIPLALQVL---IDCGKTPP--EGFKCPAS----------------
Mt1         AKDTCPIDT-LKLGACVDLL------GGLVHIGLGDPVV------NKCCPVLQGLAEIEAA-ACLCTTLKLKLLNL-NIYVPLALQLL---LTCGKTPP--PGYTCSL-----------------
Mt2         GTASCPRDA-LKLGVCANVL------NGLLNVTLGQPPV------TPCCTLLNGLVDLEAA-VCLCTALKANILGI-NLNLPISLSLLL--NVCSKQAP--RDFQCY------------------
Mt4         SSGTCPRDA-LKLGVCANVL------SGLLNLTLGKPPV------TPCCSLLNGLVDLEAA-ACLCTALKANILGI-NLNLPISLSLLL--NVCSRKVP--HDFQCA------------------
Mt3         TSQKCPSDT-LKLGVCADVL-------GLVNVIVGSPAS------SKCCALLQGLVDLDAA-ICLCTAIKANVLGI-NLNVPITLSLLL--SACEKSVP--SGFQCS------------------
Mt5         KNPTCPRDT-IKFGVCADVL-------GLINVELGKPPK------TPCCSLIDGLANLEAA-VCLCTALKANVLGI-NLNLPINLSLVL--NYCGKGVP--KGFVCA------------------
Mt7         KHPTCPRDT-IKFGVCADVL-------GLINVELGKPPK------TPCCSLIDDLANLEAA-VCLCTALKANVLGI-NLNLPINLSLVL--NYCGKGVP--KGFVCA------------------
Mt11        --PTCPRDT-IKSGVCADVL-------GLINVELGKPPK------TPCWSLIDGLANLEAA-VCLCTALKANVLGI-NLNLPINLSLVL--NYCGKGVP--KGFECA------------------
Mt12        STKSCPRDA-LKLGVCANLL------NGPIGAVIGSPPE------HPCCSILEGLVDLEVA-VCLCTAIKANILGI-DINIPISLSLIL--NACEKTPP--TDFQVLLIKVL-------------
Mt13        KHPTCPRDT-IKFGVCADVL-------GLINVELGKPPK------TPCCSLIDDLANLEAA-VCLCTALKANVLGI-NLNLPINLSLVL--NYCGKGVP--KGFVLRLI----------------
Mt6         PKGQCPKDT-LKLGVCADLL------GLVNVVIGNPPSG------SKCCALIKGLADLEAA-LCLCTALKANVLGI-NLNVPITLSLLL--SACQKTVP--PGFQCP------------------
Mt9         PKGTCPIDA-LKLGVCANLL-------NLVKVKLGSPPT------LPCCSLIQGLADLEAA-ACLCTALKAKVLGL-HLDVPISLSVIL--NNCGRNN---SGFKCT------------------
Mt8         PQGHCPKDT-LKLGVCADVL-------GLVNVVVGNPAS-----GSNCCAIIKGLADLDAA-LCLCTAIKANVLGI-NLNVPLTLTWIL--GACQKTIP--PGFQCA------------------
Mt14        PPQNCNLLN---LNICAKVL----------NNVVGLNPR-----NNRCCTLISGLVDLDAA-VCVCAALKANIIGI-SVNINADLKIIL--NSCGVNTP--AGFTCRR-----------------
Os04g52250  PTGKCPVDT-LKLLACVDAL------NGLVHAVVGATAG------DTCCPLLSGVADLDAA-LCLCTAIKAKALGL-SLVLPVAISVLV--NDCGKYVP--SDFQCPSTDPPYI*----------
Os06g07220  ATKTCPIDA-LKLNACVDVL------GGLIHLVIGQKAR------AKCCPLVQGVADLDAA-LCLCTTIRARLLNI-NIYLPVALELL---ITCGKHPP--PGFKCPPLYGA*------------
Os04g52260  PTGKCPVNT-LKLLACVDAL------NGLVHAVVGAKAS------DTCCPLLSGVADLDAA-LCLCTAIKAKALGV-SLVLPVAISVLV--NECGKHVP--SSFQCPS*----------------
Os06g43600  ATQRCPVDS-LKIGACVDLL------GGLVHVGIGDPVV------NKCCPLLEGLVELEAA-VCLCTTIRLKLLNI-NIYLPLALQLL---LTCGKNPP--PGYTCSI*----------------
Os03g01310  GGNKCPIDA-LKLGVCANVL-------NLLKLKVGVPAS------EECCPLLGGLVDLDAA-VCLCTAIKANVLGI-NINVPVDLVLLL--NYCHKTCP--SDFSCPLI*---------------
Os10g40480  RHGRCPIDA-LKLRVCTNVL------NGLVGVKIGAGP-------DDCCPLLSGLADLDAA-VCLCTAVKANVLGM-KLNLAVDLSLIL--NKCGKICP--SDFTC*------------------
Os03g01320  GGGRCPIDT-LKLGVCANVL------NGLINVQLGTPPR------QPCCSLIQGLADLEAA-VCLCTALRANILGI-NLNLPINLSLLV--NYCGRSVP--SGFQCSN*----------------
Os03g01300  GGNKCPIDA-LKLSVCANVL-------NLLKLKIGVPES------EQCCPLLGGLVDLDAA-VCLCTAIKANILGI-NLNIPVDLSLLL--NYCHKTCP--SDFTCPL*----------------
Os10g40440  GHGRCPIDA-LKLRVCANVL------NGLVGVKIGAGP-------DDCCPLLSGLADLDAA-VCLCTAIKANVLGI-INLNIPVDLSLIL-NNCGKICP--SDFTC*------------------
Os02g44310  AFGRCPRDA-LKLGVCANVL-------GLIKAKVGVPPA------EPCCPLLEGLVDLEAA-VCLCTAIRGNILGI-NLNLPIDLSLIL--NYCGKTVP--TGFKC*------------------
Os04g46810  AFGKCPRDA-LKLGVCANVL-------GLIKAKVGVPPA------EPCCPLLEGLVDLEAA-VCLCTAIRGNILGI-NLNLPVDLSLIL--NYCGKRVP--TGFKC*------------------
Os04g46820  AFGKCPRDA-LKLGVCANVL-------GLIKAKVGVPPA------EPCCPLLEGLVDLEAA-VCLCTAIKGNILGI-NLNLPVDLSLIL--NYCGKRVP--TGFKCF*-----------------
Os10g40520  STGSCPRDA-LKLRVCANVL-------GLVKAKVGAVAP-----YEPCCSLLDGLVDLDAA-VCLCTAVKANVLGI-KLDLPVDLSLIL--NNCGKICP--SDFKCVH*----------------
Os10g40430  GHGRCPIDA-LKLRVCANVL------NGALGVNVGHGPY-------DCCPLLAGLADADAA-VCLCTAVKANVLGV-NLNVPVELKLIL--NKCGKTCP--SDFTC*------------------
Os10g40614  WYGKCPTDA-LKLGVCANVL-------DLIKAKAGVPAT------EPCCPLLNGLVDLEAA-VCLCTAIKANVLGI-NLNLPIHLSLIL--NFCGKGVP--TGFMCS*-----------------
Os10g40530  GGSSCPRDA-LKLHVCANVL-------GLVKAKVGAVSP-----YEPCCSLLDGLVDLDAA-VCLCTAIKANVLGI-KLNLPIDLSLIL--NNCGKICP--SDYQCVH*----------------
Os10g40510  GAGSCPRDA-LKLHVCANVL-------GLVKAKIGAVAP-----YEPCCSLLDGLVDLDAA-VCLCTAIKANVLGL-NLNIPIDLSLIL--NNCGKICP--SDYQCA*-----------------
Os10g20890  DHGRCPIDA-LKLRVCANLL------NGLIGVKIGRGP-------DDCCPLLAGIADLDAA-VCLCTALKANVLGL-INLNLPVDLSIIL-NKCGKNYP--SGFTC*------------------
Os10g40460  RHGHCPIDA-LKLRVCANVL------NGLVGVKIGAGP-------NECCSLLQGIADLDAA-VCLCTAVKANVLGI-NLNLPVDLSLIL--NKCNKIYP--SGFTC*------------------
Os10g40470  RHGHCPIDA-LKLRVCANVL------NGLVGVKIGAGP-------NECCSLLQGIADLDAA-VCLCTAVKANVLGI-NLNLPVDLSLIL--NKCSKIYP--SGFTC*------------------
Os10g20830  EHGRCPINT-LKLRVCANVL------NGLVDAKIGHGT-------DDCCSLLSGIADLDAA-VCLCTAVKANVLGI-RVNLPVDLSIML--NKCGKTCP--SDFTC*------------------
Os10g20840  EHGRCPINA-LKLRVCANVL------NRLVDVKIGHGP-------DDCCSLLSGIADLDAA-VCLCTAVKANVLGI-RVNLPVDLSLIL--NKCGKSCP--SDFTC*------------------
```

```
Os04g46830  YHNKCPVNT-LKFGACADVL-------GAISGEVGQVP------AQPCCSLISGLADLEAA-VCLCTAIKANVLGV-VVNIPVKLSLLV--NYCGKCVP--SGYTCA*-----------------
Os06g01580  PAGKCPINT-VKLGVCADVL------DGLIHASTPPK--------EPCCPLIAGLADLDAA-VCVCLAINANLLGL-NLDVPVDLSLLL--NYCGCKLP--AGFNTRGGCWRGGGG---------
Os02g44320  YNTKCPKNA-LKFAACADVL-------GLVSAEVGQPP------YEPCCGVLGGLADLEAA-VCLCTAIKANVLGI-TLDIPVKLSLLV--NYCGKNVP--SGFICA*-----------------
Os10g40420  AGAKCPFDA-LKLAACADVLGGGGGGGGLLNLGHLLGNSSPSSSGEQCCGLLAGLADVDAA-VCLCTALRANVLGL-VGVEPHVQLSVLV-NRCSRKLP--NGFQCSSN*---------------
Os04g55170  VNPFCPWDA-VKFGACAGVL-------GVVGVQAGAHL------GSKCCALVDGLAAAEAA-ACFCTTIKESVLGI-PTEWTVGVSVLV--STCKTELP--DGFKCV*-----------------
Os10g09920  TGGTCPINV-LNLAVCANVL-------SLNVPSS------------QCCTLLQGLADLDAA-LCLCAALKANILGV-INVDVLVDVTLIL-NSCNRTCP--PGFTCPL*----------------
Os03g14654  PPPKCPLAL-INLNACISV--------GLGNPLLNQ----------ACCSQLSSLPSDTAA-VCLCEAIKVNALVN-LKVKIPDIL-----KVCGKV----SAVVCV*-----------------
Os03g50960  RGNPCPTSALADLKVCADVLVL----LKLKINVPASQ---------QCCPLLGSLVNLDAA-ACLCAAIRLSVLGI-PVNLPLDVPLVL--NYCGRNASA-AGANCS*-----------------
Os03g26800  PAVDCTAAEALKVGACLDYVTP-----GNPPRSQPSK---------ACCGEVKGVLKDIAGVGCLCAAISTHALPL-PINATRVLHLP---AACGADAS--AFTMCLGQSTYFDLLLL*------
```
