# Supplementary material for: Analysis of the hybrid proline-rich protein families from seven plant species suggests rapid diversification of their sequences and expression patterns
Source: BMC Genomics. 2007 Nov 12;8:412. doi: 10.1186/1471-2164-8-412 (PMC2216038; doi:10.1186/1471-2164-8-412)

A phylogenetic tree of HyPRP sequences rooted using the *Arabidopsis thaliana* AtLTP2 (At2g38530) sequence as outgroup. NJ (top) and ML (bottom) bootstrap values above 50 % are shown at branches of the tree (constructed by the NJ method using Treecon). The outgroup sequence was added manually to the alignment of HyPRP sequences, using the invariant cysteine positions and published 8CM domain alignments as a guide. The tree was then calculated from a subset of HyPRP sequences from Figure 4, selected in order to keep representatives of all branches with a significant support in trees constructed using both NJ and ML method; in addition, several sequences whose inclusion would have introduced gaps in the alignment and thus reduced the amount of usable data have been excluded. HyPRPs with long N-terminal domains are marked in **bold**, the clade of C-type HyPRPs is boxed. Bootstrap values in blue correspond to an otherwise identical tree calculated after omission of the outlier sequences with long N-terminal domains (also shown in blue, ML values from only 100 replicates), indicating that these probably rapidly evolving sequences were responsible for most of the uncertainty in the delimitation of the C-type clade.

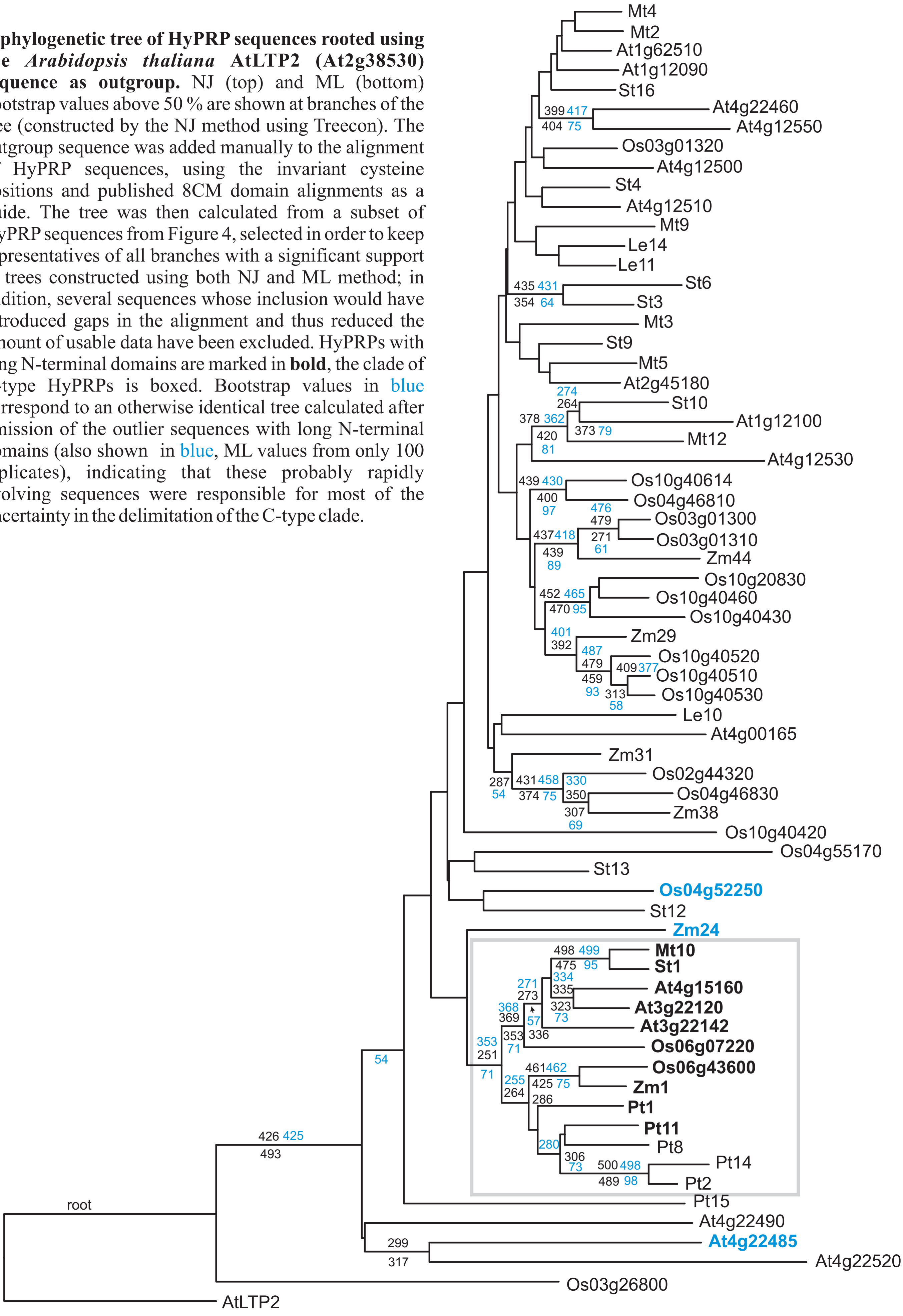

Supplement: Additional file 4 — A phylogenetic tree of HyPRP sequences rooted using Arabidopsis thaliana AtLTP2 as an outgroup. LTP-rooted phylogenetic tree of representative sequences of all significantly supported branches from Fig. 3. The effects of the omission of three possible outlier C-type sequences on the bootstrap support of the C-type branch are indicated. [file 1471-2164-8-412-S4.pdf]
